# Microbial-derived peptidases are altered in celiac disease, non-celiac gluten sensitivity, and functional dyspepsia: a systematic review and re-analysis of the duodenal microbiome

**DOI:** 10.1080/19490976.2025.2500063

**Published:** 2025-05-09

**Authors:** Jennifer C. Pryor, Cheenie Nieva, Nicholas J. Talley, Guy D. Eslick, Kerith Duncanson, Grace L. Burns, Emily C. Hoedt, Simon Keely

**Affiliations:** a College of Health, Medicine and Wellbeing, The University of Newcastle, Callaghan, NSW, Australia; b National Health and Medical Research Council (NHMRC), Centre of Research Excellence in Digestive Health, The University of Newcastle, Newcastle, NSW, Australia; c Immune Health Research Program, Hunter Medical Research Institute, New Lambton Heights, NSW, Australia

**Keywords:** Celiac disease, gluten, NCWS, NCGS, DGBI, microbiota, functional dyspepsia

## Abstract

Dietary gluten triggers symptoms in patients with gluten-related disorders (GRDs) including celiac disease (CeD), non-celiac gluten sensitivity (NCGS), and subsets of patients with functional dyspepsia (FD). The gastrointestinal microbiota is altered in these patients when compared to healthy individuals. As the microbiota is crucial for the hydrolysis of gluten, we hypothesized that the capacity of the microbiota to digest gluten is reduced in these conditions. We systematically reviewed and re-analyzed published datasets to compare gastrointestinal microbiomes of GRD patients and identify signals explaining gluten responses. A systematic search of five databases was conducted to identify studies where the microbiota of CeD, NCGS, or FD patients was analyzed by 16S rRNA amplicon or shotgun metagenomic sequencing and compared to control populations. Where available, raw duodenal microbiota sequence data were re-analyzed with a consistent bioinformatic pipeline. Thirty articles met the inclusion criteria for this systematic review. Microbiota diversity metrics were not impacted by the diseases; however, genera including *Streptococcus*, *Neisseria*, and *Lactobacillus* were commonly altered in GRD patients. Re-analysis of duodenal 16S rRNA data was possible for five included articles but did not identify any consistent differentially abundant taxa. Predicted functional analysis of the microbiome revealed that peptidases including aminopeptidase, proline iminopeptidase, and Xaa-Pro dipeptidase are altered in CeD, NCGS, and FD, respectively. These microbial-derived peptidases hydrolyze bonds in proline-rich gluten peptides. While the gastrointestinal microbiota in patients with GRDs differ from controls, no distinct phenotype links them. However, alterations to the predicted functional capacity of the microbiome to produce gluten-hydrolyzing enzymes suggest that inappropriate digestion of gluten by the microbiome impacts host responses to dietary gluten in these conditions. These findings have implications for therapeutic management of GRDs, as treatment with gluten-degrading enzymes or tailored probiotics could improve disease outcomes by enhancing gluten digestion into non-reactive peptides.

## 1. Introduction

Wheat is a key food source for approximately 40% of the world’s population, and gluten is its primary protein constituent.^1^ Despite this, avoidance of wheat and adherence to a gluten-free diet (GFD) is increasing globally, with the prevalence of GFD adherence ranging between 0.5% and 24% of the general population.^2–4^ This is likely due to the rise in gluten-related disorders (GRDs) and lifestyle preferences.^5,6^ GRDs refer to a heterogeneous array of conditions in which gluten has been identified to trigger symptoms. Coeliac disease (CeD) is the most well characterized of the GRDs, affecting up to 1% of the population globally.^7^ It is an autoimmune disease distinguished by specific serological markers including elevated transglutaminase (tTG) and deamidated gliadin antibodies.^8^ CeD has a distinct histopathological profile, defined by small intestinal villous atrophy resulting from a severe immune response upon gluten ingestion.^9^ This causes a range of gastrointestinal and extra-intestinal symptoms which can only be alleviated by the strict avoidance of dietary gluten.^10^ Similarly, patients with non-celiac gluten/wheat sensitivity (NCGS) experience a variety of symptoms following gluten consumption; however, in these patients, CeD has been excluded either serologically or histologically.^11^ No well-defined mechanism has been established for the pathophysiology of NCGS; however, recent evidence has identified subtle signs of inflammation, namely altered recruitment of eosinophils, mast cells, and innate lymphoid cells to the gastrointestinal mucosa of these patients.^12–14^ According to an Australian population-based study, NCGS was self-reported in 13.9% of the responders and appears to have significant overlap with some functional gastrointestinal disorders, especially functional dyspepsia (FD).^4,15^ FD reportedly has a global prevalence of 8.4%, and patients are diagnosed according to the ROME criteria based on meal-related symptoms of early satiety or post-prandial fullness, or epigastric pain or burning.^16,17^ Many people with FD report that gluten-containing foods trigger their symptoms, and self-reported NCGS was found to be significantly associated with FD diagnosis in a longitudinal cohort study.^4^ Whilst no overt pathology can be identified during endoscopy of FD patients, there is evidence of subtle inflammation within the duodenum, similar to that found in NCGS, especially elevated duodenal eosinophils.^18–20^ Due to the overlapping phenotype, it is likely that NCGS exists in at least a subset of FD patients.^21,22^

In addition to dietary gluten triggering symptoms, another key factor that links FD, NCGS, and CeD is the evidence that the microbiota is altered within the gastrointestinal tract of these patients when compared to controls. The gastrointestinal microbiota plays a crucial role in digestion and maintenance of immune and physiological homeostasis. Significant alterations to the composition of the microbiota can impact key microbial functions, and this “dysbiosis” has been implicated in a range of gastrointestinal conditions.^23,24^ Many food components, including gluten, are somewhat resistant to host-derived enzymes and require the microbiota for complete digestion.^25^ This process is key for the liberation of nutrients and to prevent inappropriate immune responses to incompletely digested foods.^26^ A study on human intestinal organoids described that microbiome-derived byproducts could modulate epithelial and cytokine responses to gluten.^27^ Gluten hydrolysis is performed by a variety of microbial species, with one study identifying more than 80 small intestinal-derived bacterial strains that could survive on gluten as the sole nitrogen source.^25^ Therefore, an altered microbiome may have an impaired ability to digest gluten, and this may impact immune and physiological responses to gluten.

Given the role of the microbiome in digesting gluten and maintaining immune homeostasis, we sought to conduct a systematic review to compare the microbiota composition in three conditions (CeD, NCGS, and FD) where gluten-based foods are reported to trigger symptoms. Methodologies to investigate the composition of the microbiota are highly variable; from targeted polymerase chain reaction (PCR) and culturing approaches to non-targeted approaches such as amplicon-based sequencing (i.e., 16S rRNA gene) or shotgun metagenomic sequencing. Whilst the non-targeted sequencing approaches minimize some bias in microbiome analysis, the large amount of variation in the sequencing and analysis approach can make it difficult to confidently draw conclusions. Thus, we additionally aimed to re-analyze raw duodenal 16S rRNA data from published FD, NCGS, and CeD datasets, using a uniform bioinformatic pipeline to minimize analysis bias. This analysis sought to identify microbial signals or functional pathways associated with GRDs.

## 2. Methods

### 2.1. Registration

This review was prepared in accordance with the Preferred Reporting Items for Systematic Reviews and Meta-Analyses (PRISMA) guidelines (Supplementary Figure S1).^28^ The systematic review protocol has been registered with the International Prospective Register of Systematic Reviews (PROSPERO), under the reference CRD42021285904.^29^

### 2.2. Search strategy

We conducted a systematic search for published articles reporting on the gastrointestinal microbiota in patients with FD, NCGS, or CeD. We electronically searched EMBASE (OVID), Medline (OVID), Cochrane Library (Wiley Online), Scopus (Elsevier), and Web of Science (Thomas Reuters) up to 30^th^ May 2024. Our search terms included: [non-celiac gluten sensitivity OR non-celiac wheat sensitivity OR gluten sensitivity OR gluten hypersensitivity OR wheat sensitivity OR non-celiac gluten intolerance OR celiac disease OR celiac sprue OR functional dyspepsia OR dyspepsia OR non-ulcer dyspepsia OR idiopathic dyspepsia] AND [microbiota OR microbiome OR gastrointestinal microbiome OR intestinal microbiota OR microbial consortia]. No language or date restrictions were utilized in the search strategy or study selection.

### 2.3. Study selection and quality assessment

Title and abstract screening was performed by two independent reviewers using the web application, Covidence (www.covidence.org). Full-text screening was similarly conducted in duplicate, and articles meeting the following criteria were included: (1) articles reported on patients with either FD, NCGS, or CeD; (2) original 16S rRNA gene sequencing or shotgun metagenomic sequencing of gastrointestinal samples was performed; (3) articles reported microbiota data for cases and an appropriate control group prior to any intervention; (4) participants were aged 18 or older. Conference abstracts, letters, editorials, case reports, reviews, and study protocols were excluded from this review. The quality of the included articles was assessed utilizing the Joanna Brigg’s Institute (JBI), a critical appraisal tool for case-control studies. This tool evaluates articles based on the selection of cases and controls, the reliability of measurements, and the handling of confounding factors.

### 2.4. Data extraction

Data manually extracted from selected articles included: year of publication, country, number and type of subjects, diet followed, sample site and type, sample collection and storage, DNA extraction technique, sequencing method, analysis pipeline, and database for taxonomy assignment. Data describing microbial diversity metrics, microbial abundance, and microbial function at the baseline compared to a comparator or control group were also extracted into Excel Spreadsheets. Where available, for studies which reported on the duodenal microbiota by 16S rRNA amplicon sequencing, raw sequence reads and associated metadata were retrieved for use in the uniform analysis.

### 2.5. Uniform analysis of raw published data

We chose to re-analyze available 16S datasets using a consistent bioinformatic pipeline to minimize variation.^30,31^ This analysis focussed on datasets pertaining to the duodenal microbiota, as there is evidence linking the pathophysiology of each of the studied conditions with the duodenum. Where available, raw sequencing data and metadata were downloaded from the NCBI Sequence Read Archive. Alternatively, when data was not available publicly, authors were contacted to request the raw sequences and metadata. Due to 16S sequence batch effects, each study was processed and analyzed separately.

Sequence data was processed with the Quantitative Insights into Microbial Ecology 2 pipeline (QIIME2 v2022.2).^32^ Primers were trimmed from the raw sequences with the Cutadapt plugin.^33^ Quality assessment, demultiplexing, and denoising were then completed using the divisive amplicon denoising amplicon 2 (DADA2) package.^34^ Taxonomy was assigned to the sequences in RStudio (v2024.04.1) with R (v4.4.0), using the DADA2 (v1.24.0) package and the SILVA reference database (v138).^35^ Eukaryotic sequences and samples with low sequencing results were excluded from the analysis. Analysis of microbial diversity was conducted with the R-package phyloseq (v1.48.0).^36^ The alpha diversity was calculated with Chao1, Shannon, and Simpson metrics, and a pairwise Wilcox test, with Benjamin Huxburg adjusted p-value, was performed to assess significance. Beta diversity Bray-Curtis distance significance was assessed by permutational multivariate analysis of variance (PERMANOVA) using Adonis2 in the vegan package (v2.6–4). To identify differentially abundant taxa between groups, multiple approaches were applied as there is no clear consensus on the best approach.^37^ This included linear discriminant analysis effect size (LEfSe), EdgeR, and Analysis of Compositions of Microbiomes with Bias Correction 2 (ANCOM-BC2) within the microbiomeMarker package (v1.2.2).^38^ Functional capabilities of the sequenced microbiota were predicted with phylogenetic investigation of communities by reconstruction of unobserved states 2 (PICRUSt2)^39^ within QIIME2 (v2021.11) and analyzed using the R package phyloseq (v1.48.0). For all data, *p* < 0.05 was considered significant.

### 2.6. Phylogenetic tree

A phylogenetic tree was constructed to compare bacterial peptidases reported to be associated with gluten digestion. Following a literature search, protein sequences of peptidase gluten hydrolyzing capacity were collated from the NCBI protein database.^40–42^ The sequences were largely derived from Klebsiella pneumoniae, unless unavailable then a suitable alternative was selected. Multiple sequence alignment was performed in MEGA (v11.0.13) by ClustalW. The Neighbor-Joining method was utilized to construct the phylogenetic tree with 1000 bootstrap replicates.

### 2.7. Data availability

All data utilized are available from publicly available, published studies.

## 3. Results

Of the 5,961 records identified from the systematic search, 2,658 articles were excluded as duplicates (Supplementary Figure S2). The remaining 3,303 records were subject to title and abstract screening which identified 125 articles to assess for inclusion by full text review. Reasons for exclusion following full text review included articles containing only abstracts or editorials, articles focusing on pediatric populations, and studies lacking an appropriate control cohort. Articles were also excluded if the microbiome was not reported on prior to an intervention or if the microbiome was analyzed by methods other than 16S rRNA gene or shotgun metagenomic sequencing (Supplementary Figure S2).

Thirty articles that detailed gastrointestinal microbiota sequencing of FD, NCGS, or CeD patient samples as they were compared to controls^43–73^ were included in this systematic review. Five of these articles had available 16S data for the duodenal microbiota, along with sufficient metadata for re-analysis using a consistent bioinformatic pipeline.^43,45,51,64,65^ Across the 30 included studies the median JBI quality score was 8/10 (range 4–9, standard deviation = 1.42), indicating that the methodology of the articles was of high quality with minimized risk of bias (Table 1).

**Table 1.** Characteristics of studies investigating the gastrointestinal microbiota in celiac disease, non-celiac gluten sensitivity, and functional dyspepsia.

Study	Country	Year	Diagnosis (n)	Baseline Diet (n)	Age (mean ±SD)	Sex, male n (%)	Control/Comparator (n)	Age (mean ±SD)	Sex, male n (%)	Study aim	JBI Quality Score
Bodkhe et al	India	2019	CeD (23)	Normal (23)	23.4 ± 9.5	10 (43%)	Healthy FDR (15) DC (24)*	FDR: 31.6 ± 10.8 DC: 30.6 ± 12.3	FDR: 6 (40%) DC: 22 (92%)	To assess differences in the microbiota of CeD patients and their first-degree relatives.	8
Constante et al	Canada	2022	CeD (24)	Normal (24)	40.3 ± 15.94	10 (42%)	Healthy (30) Hospital controls (11)	40.6 ± 14.7	16 (39%)	To compare the microbiota profiles of CeD patients and controls at different gastrointestinal sites.	8
D’Argenio et al	Italy	2016	CeD (26)	Normal (20) GFD (6)	aCeD: 38 ± 12 GFD CeD: 39 ± 11	aCeD: 2 (10%) GFD CeD: 1 (17%)	DC (15)*	42 ± 16	2 (13%)	To determine CeD-associated duodenal dysbiosis and the role of dysbiosis in CeD pathogenesis.	8
Francavilla et al	Italy	2023	CeD (66)	Normal (3) GFD (63)	GFD CeD: 42 ± 14.5 aCeD: 46.5 ± 13.9	GFD CeD: 15 (24%) aCeD: 0	Healthy (66)	40.8 ± 14.3	15 (23%)	To explore how specific patterns and microbial profiles are impacted by length and adherence to GFD.	9
Iaffaldano et al	Italy	2018	CeD (36)	Normal (14) GFD (22)	GFD CeD: 32 ± .4^†^ aCeD: 26 ± .4^†^	GFD CeD: 4 (19%) aCeD: 6 (43%)	Healthy (20)	30±.4^†^	4 (30%)	To characterize the oropharyngeal microbiota of CeD patients and evaluate its association with the duodenal microbiota	9
Lopetuso et al	Italy	2020	CeD (4)	GFD (4)	41.5 ± 6.9^†^	2 (50%)	Healthy (60)	31.0 ± 2.0^†^	34 (57%)	To explore the role of Akkermansia muciniphila as a marker of dysbiosis and disease state in a large disease cohort compared to controls.	7
Nistal et al	Spain	2012	CeD (10)	Normal (5) GFD (5)	aCeD: 31.4 GFD CeD: 18.8	aCeD: 3 (60%) GFD CeD: 2 (40%)	Healthy (5)	29.2	2 (40%)	To characterize duodenal differences in bacteria between healthy and CeD adults and children.	5
Nistal et al	Spain	2016	CeD (9)	Normal (9)	Not described	Not described	Hospital controls (9)	Not described	Not described	To characterize the composition of the duodenal microbiota of untreated CeD and controls.	5
Palmieri et al	Italy	2022	CeD (46)	GFD (46)	38.6 ± 18.2	18 (39%)	Healthy (30)	46 ± 8.0	17 (57%)	To highlight the role of the microbiota in CeD by analyzing a well-characterized cohort of treated CeD patients.	4
Panelli et al	Italy	2020	CeD (46)	Mediterranean (13) Gluten-free Mediterranean (33)	aCeD: 35 ± 6 GFD CeD: 37 ± 6 rCeD: 53 ± 15	aCeD: 2 (15%) GFD CeD: 9 (31%) rCeD: 0	FD (31)* pCeD (6)	FD: 44 ± 17 pCeD: 41 ± 14	FD: 7 (23%) pCeD: 3 (50%)	To characterize the mucosal microbiota of CeD patients and compare to controls. To assess whether salivary samples mirrored the microbial profile of the mucosa better than stool.	8
Rawson et al	USA	2023	CeD (12)	GFD (12)	35–44	2 (17%)	Healthy (9) GFD Healthy (7)	25–34	Healthy: 1 (14%) GFD Healthy: 3 (44%)	To compare the microbiome of people with CeD on a GFD with those on a GFD without CeD, and a control group (without CeD and a GFD).	7
Serena et al	USA	2019	CeD (18)	Normal (10) GFD (8)	Not described	Not described	Healthy (10)	Not described	Not described	To evaluate if CeD patients have altered blood microbiome and if these changes relate to gut microbiota composition and loss of tolerance to gluten.	7
Shi et al	China	2022	CeD (30)	Normal (30)	40.9 ± 12.4	7(23%)	Healthy (30)	39.8 ± 11.2	7(23%)	To analyze the fecal microbiota composition and metabolome characteristics of CeD patients, and screen for biomarkers.	9
Slager et al	Netherlands	2024	CeD (153)	GFD (153)	30	55 (36%)	Healthy (765)	30	315(41%)	To investigate the microbiota composition and function in a large sample of CeD patients and controls.	8
Tian et al	USA	2017	CeD (29)	GFD (29)	GFD CeD: 36.2 ± 17.0 rCeD: 54.1 ± 13.5	GFD CeD: 3 (14%) rCeD: 3 (378%)	Healthy (20) FGIDs (12)*	Healthy: 35.4 ± 15.7 FGIDs: 40.2 ± 14.4	Healthy: 5 (25%) FGIDs: 4 (33.3%)	To investigate oral microbial profiles and salivary enzymatic activity of CeD patients and controls.	9
Garcia-Mazcorro et al	Mexico	2018	CeD (6) NCGS (12)	Normal (18)	CeD: 46 ± 18.2 NCGS: 30 ± 11.0	CeD: 0 NCGS: 1 (8%)	Healthy (12)	35 ± 14.7	6 (50%)	To investigate the gut microbiota composition and predicted functional profile in Mexican patients with gluten related disorders.	7
Nobel et al	USA	2021	CeD (9) NCGS (8)	GFD (17)	GFD CeD: 57.9^‡^ GFD NCGS: 51.9^‡^	GFD CeD: 4 (44%) GFD NCGS: 0	Healthy (8)	40.4^‡^	4 (50%)	To investigate the impact of gluten exposure on the gut microbiota composition of CeD and NCGS and compare to controls.	9
Nylund et al	Finland	2020	CeD (19) NCGS (10)	GFD (29)	GFD CeD: 51^‡^ GFD NCGS: 34^‡^	GFD CeD: 4 (21%) GFD NCGS: 1 (10%)	Healthy (14)	34^‡^	6 (43%)	To evaluate the effect of oat consumption on diet and gut well-being among adults with gluten-related disorders.	8
Dieterich et al	Germany	2019	NCGS (19)	Western standard diet (19)	33.8 ± 11.9	4 (21%)	Healthy (10)	32.8 ± 10.9	3 (30%)	To study the effect of a low FODMAP diet and GFD on symptoms, mucosal inflammation, and microbiota composition of NCGS patients and controls.	9
Shah et al	Australia	2022	FD with NCGS (20)^a^ FD (20)	Normal western diet (40)	FD w NCGS: 53.8 ± 15.0 FD: 49.6 ± 15.1	FD w NCGS: 8 (40%) FD: 10 (50%)	Hospital Controls (18)	60.0 ± 11.2	8 (44%)	To investigate if FGID patients have an augmented response to a standardized nutrient challenge. To investigate if FGID patients with and without NCGS differ in immune activation and microbiota composition	9
Cervantes et al	USA	2021	Dyspepsia (25)	Not defined	Not described	Not described	Achalasia (11)	Not described	Not described	To compare the microbiota composition of the duodenum, the stomach and saliva in patients with symptoms of dyspepsia compared to achalasia patients used as controls.	8
Fukui et al	Japan	2020	FD (11)	Not defined	56^‡^	6 (55%)	Healthy (7)	52^‡^	4 (57%)	To evaluate the microbiota associated with FD by brushing of mucosal surfaces under endoscopy and clarifying the correlation between FD and microbiota.	9
Igarashi et al	Japan	2017	FD (24)	Not defined	44^‡^	12 (50%)	Healthy (21)	42^‡^	14 (67%)	To analyze the gastric fluid microbiota between patients with FD and healthy controls, and to assess the effect of probiotics on the microbiota	9
Kim et al	South Korea	2023	FD (12)	Normal (12)	43.1 ± 3.5^†^	3 (25%)	Healthy (15)	35.6 ± 2.7^†^	4 (27%)	To understand the interconnection of FD symptoms, nutrition, duodenal inflammation and permeability, and the oral and gut microbiota	9
Kovaleva et al	Russia	2023	FD (60)	Normal (60)	33.75 ± 9.52	23 (62%)	Healthy (20)	39.13 ± 15.20	5 (25%)	To evaluate and compare the gut microbiota composition and SCFA profiles in patients with combined IBS-D and FD with those in healthy control subjects.	8
Liu et al	China	2021	FD (21)	Not defined	38.0 ± 15.8	9 (43%)	Healthy (12)	25.0 ± 6.2	7 (58%)	To study changes to oral flora in FD and to compare a range of oral factors between FD and controls.	6
Nakae et al	Japan	2016	FD (44)	Not defined	42.5^‡^	22 (50%)	Healthy (44)	41.5^‡^	30 (68%)	To identify the bacterial biomarkers of FD within gastric fluid and assess the effects of LG21 probiotic yogurt.	9
Shanahan et al	Australia	2022	FD (56)	Normal (56)	47^‡^	27 (48%)	Hospital controls (30)	59^‡^	15 (50%)	To examine the duodenal, mucosal microbiota in FD and integrate with upper gut function, symptoms and habitual dietary intake.	9
Wauters et al	Belgium	2021	FD (47) (off PPI, *n* = 28; on PPI, *n* = 19)	Normal (47)	FD off PPI: 27 FD on PPI: 19	FD off PPI: 4 (14%) FD on PPI: 5 (26%)	Healthy (30)	27	9 (30%)	To characterize the duodenal mucus- and epithelium-associated microbiota of FD patients and controls, and to assess the effect of PPI therapy on the duodenal microbiota.	9
Zheng et al	China	2022	FD (20)	Not defined	36.6 ± 14.0	3 (15%)	Healthy (5)	28.8 ± 9.0	1 (20%)	To study the differences of duodenal microflora and its function in patients with FD and healthy controls.	6

*This control group contains functional dyspepsia patients.

^†^mean ± Standard Error of the Mean.

^‡^median.

^a^This group contains two patients with IBS who do not meet the symptom criteria for FD.

aCeD, active celiac disease; CeD, Celiac Disease; DC, Disease controls; FD, Functional dyspepsia; FDR, First-degree relative; FGID, Functional gastrointestinal disorder; FODMAP, fermentable oligosaccharides, disaccharides, monosaccharides, and polyols; GFD, Gluten free diet; HC, hospital controls; NCGS, non-celiac gluten sensitivity; PPI, Proton-pump inhibitors; pCeD, potential Celiac Disease; rCeD, refractory celiac disease

### 3.1. Study characteristics

From the selected articles, 18 described the gastrointestinal microbiota of patients with CeD^43,45,46,49,51,52,55,57–63,66,67,72,73^; 11 reported on FD^44,50,53,54,56,64,65,68–71^; and five described NCGS^47,51,59,60,64^ (Table 1). The included studies were conducted across broad geographic regions, with 13 articles reporting on patients in Europe,^46,47,49,52,55,57,58,60–62,68,71,73^ eight in Asia,^43,50,53,54,56,66,69,70^ seven in North America,^44,45,51,59,63,67,72^ and two in Australia.^64,65^ The articles were published between 2012 and 2024, with the median year of publication being 2021.

Across all studies a total of 566 patients with CeD were described, 168 of these had active disease (aCeD); 386 patients were treated with a GFD (GFD CeD); and 12 were on a GFD with refractory symptoms (rCeD). A total of 69 NCGS patients were included, with 18 on a GFD (GFD NCGS) and 51 active NCGS patients (aNCGS) consuming a non-restricted diet. Finally, 371 patients with FD were included in this systematic review. Among the included FD patients, the 25 patients identified in the study by Cervantes et al. were labeled as ‘dyspepsia’.^44^

Control and comparator cohorts for the included studies varied somewhat. Most papers compared cases to healthy controls, with 1250 healthy participants included. Fifteen healthy first-degree relatives (FDRs) were used as a comparison of the pre-disease state to CeD patients in Bodkhe et al.^43^ Four studies compared cases to patients described as hospital controls.^45,58,64,65^ Hospital controls were defined as patients attending endoscopy who had normal CeD serology, no abnormalities on endoscopy, no auto-immune conditions, and minimal or no gastrointestinal symptoms. The 68 hospital controls included were chosen due to the ethical difficulties in sampling the gastrointestinal tract of healthy patients via endoscopy.^45,58,64,65^ Similarly, a further 93 disease controls across five studies were included, largely due to the ease of sampling.^43,44,46,62,67^ This included 11 patients diagnosed with achalasia chosen by Cervantes et al.,^44^ as a comparison to FD given the absence of documented pathology below the gastroesophageal junction in achalasia. Additionally, four disease control cohorts included FD patients, with the control cohort for CeD patients in Panelli et al.,^62^ consisting entirely of FD patients. FD patients were included as controls in studies of CeD largely due to the lack of overt pathology found within the gastrointestinal tract of FD patients. Furthermore, Panelli et al.,^62^ included patients with potential CeD (pCeD), defined as having positive CeD serology but no villous atrophy upon endoscopic examination. For the sake of this systematic review, we included pCeD as a comparator group.

The composition of the microbiome can be greatly influenced by diet and medications, and these factors should be considered when interpreting results of microbiome studies. To control for some of the external influences on the microbiome, 19 of the included articles excluded participants treated with antibiotics prior to sampling, while 10 studies excluded patients taking protein pump inhibitors (PPIs). Five articles acknowledged that their cohorts included participants currently on PPI therapy. Most of these articles were from studies on FD patients, which are to be expected, as PPIs are a common treatment for symptom management in FD. All of the included studies on CeD and NCGS described the diet of participants as either normal, gluten-containing, or gluten-free, with one study further defining the diet as Mediterranean. One study each examining FD and NCGS described the diet of participants as Western; while a further four studies on FD reported that participants were consuming their normal diets. Six of the included articles on FD did not report on the diet of participants prior to baseline sampling (Table 1).

### 3.2. Microbiota sampling and assessment methods

The microbiota was sampled from stool in 16 of the included studies^43,45,47,49,51,55,59–63,66,70–73^; 15 studies sampled the duodenum^43–46,50–52,57,58,62,64,65,68–70^; seven assessed the oral or esophageal microbiota^44,50,52,54,62,67,70^; and four studies assessed the gastric compartment^44,50,53,56^ (Table 2). Following the collection, samples were stored at −80°C in 18/30 studies. Bead beating or mechanical lysis was a common pre-processing step for microbial DNA extraction and was described in 7/30 articles. Commercial DNA extraction kits, notably those sourced from Qiagen, were the preferred choice to prepare samples for sequencing. Three studies, however, employed the manual extraction methods of phenol–chloroform extraction^45,52^ or the cetyltrimethylammonium bromide method.^69^

**Table 2.** Microbiota sequencing methodologies utilized in studies examining celiac disease, non-celiac gluten sensitivity, and functional dyspepsia.

Diagnosis	Study/Year	Specimen (location)	Sample Collection/Storage conditions	Extraction method	Sequencing approach	Platform	Variable regions amplified	Analysis pipeline (version)	Taxonomic database (version)
CeD	Bodkhe et al.^43^	Stool Biopsy (duodenum)	Not described	Stool: QIAamp fast DNA stool Mini Kit (Qiagen) Biopsy: DNeasy Blood and Tissue kit (Qiagen)	16S	Illumina MiSeq	V4	Cutadapt (1.18) DADA2 (1.6.0)	Human Intestinal 16S rRNA gene reference database (HITdb 1.00)
CeD	Constante et al.^45^	Stool Biopsy (duodenum) Aspirate (duodenum)	Stool: anaerobic atmosphere generation bags (Sigma-Aldrich); −80°C Biopsy: collected with sterile forceps; −80°C Aspirate: sterile waterjet used to slightly dislodge mucus, followed by immediate collection; −80°C	Mechanical and enzymatic lysis followed by phenol-chloroform DNA extraction	16S	Illumina MiSeq	V3 (or V34 for some stool samples)	Cutadapt R (4.1.1) DADA2	SILVA reference database (138.1)
CeD	D’Argenio et al.^46^	Biopsy (duodenum)	Biopsies were collected under sterile conditions and cooled in dry ice; −80°C	QIAamp DNA Mini Kit (Qiagen)	16S	Roche 454 Genome Sequencing system	V4-V6	QIIME (1.9.1)	Greengenes database (13.8)
CeD	Francavilla et al.^49^	Stool	Collected in nucleic acids stabilizing solution (Norgen Biotek), vortexed, and aliquoted within a few hours of collection; −80°C	DNeasy PowerSoil Pro Kit (Qiagen)	Shotgun metagenomic sequencing	Illumina NovaSeq6000	Not applicable	MetaPhlAn3	mpa_v30_CHOCOPhlAn_201901
CeD	Iaffaldano et al.^52^	Swab (oropharyngeal) Biopsy (duodenum)	Swabs collected in Liquid Amies Elution Swab (Eswab) by touching the back wall of the oropharynx only. Swabs were cooled in 10% glycerol on dry ice, −80°C	Phenol-chloroform DNA extraction	16S	Illumina MiSeq	V4-V6	QIIME (1.9.1)	Greengenes database (13.8)
CeD	Lopetuso et al.^55^	Stool	Three stool samples were collected from each case and one sample from each control	QIAamp DNA Stool Mini Kit (Qiagen)	16S	Illumina MiSeq	V3-V4	QIIME (1.8)	Greengenes database (13.8)
CeD	Nistal et al.^57^	Biopsy (duodenum)	Stored in saline solution; −80°C	NucleoSpin Tissue XS kit (Macherey-Nagel)	16S	Pyrosequencing	V6-V8	BioEdit MEGA (4.0.1)	Ribosomal Database Project (release 10)
CeD	Nistal et al.^58^	Biopsy (duodenum)	Stored in saline solution; −80°C	Mechanical lysis & DNeasy Blood and Tissue kit (Qiagen)	16S	Roche Pyrosequencing Flx System	V4	Mothur (1.29)	Ribosomal Database Project
CeD	Palmieri et al.^61^	Stool	Sterile container; −20°C followed by −80°C	QIAamp PowerFecal DNA Kit (Qiagen)	16S	Illumina MiSeq	V3-V4	QIIME2 (2021.11)	Greengenes database (13.8)
CeD	Panelli et al.^62^	Stool Saliva Biopsy (duodenum)	Stool: −20°C followed by −80°C Saliva: direct spitting into a sterile plastic tube; −80°C Biopsy: snap frozen, −80°C	Stool: QIAamp DNA Stool Mini kit (Qiagen) Saliva: QIAamp DNA Blood Mini Kit (Qiagen) Biopsy: DNeasy Blood and Tissue Kit (Qiagen)	16S	Illumina MiSeq	V3-V4	ad hoc bioinformatics pipeline built under R USEARCH (10.0.240)	Greengenes database (13.8)
CeD	Rawson et al.^72^	Stool	Collected by patient and frozen; −80°C	QIAamp PowerFecal DNA Kit (Qiagen)	16S	Illumina MiSeq	V3-V4	Nephele (2.10.0.) QIIME 2	Greengenes database
CeD	Serena et al.^63^	Stool	Frozen	Qiagen DNeasy powersoil extraction kit (Qiagen)	16S	Illumina MiSeq	V4	QIIME (2018.2.0)	Greengenes database (13.8)
CeD	Shi et al^66^	Stool	Not described	FastDNA Spin Kit for soil (MP bio)	16S	Illumina MiSeq	V3-V4	QIIME2 DADA2 (1.6.0)	Ribosomal Database Project (11.5)
CeD	Slager et al.^73^	Stool	Collected by patient and frozen; −80°C long term storage	Isolation with QIAamp Fast DNA Stool Mini Kit (Qiagen), using the QIAcube (Qiagen) automated sample preparation system	Shotgun metagenomic sequencing	Illumina HiSeq 2000	Not applicable	KneadData (0.10.0) MetaPhlAn3 (3.0.1)	MetaPhlAn database mpa_v30
CeD	Tian et al.^67^	Saliva	Collected by a dentist into 50-ml centrifuge tubes on ice, vortexed, and frozen in 1 ml aliquots; −80°C	MasterPure Gram-positive DNA purification kit (Epicentre)	16S	Illumina MiSeq	V3-V4	QIIME	HOMD reference database
CeD/NCWS	Garcia-Mazcorro et al.^51^	Stool Biopsy (duodenum)	Not described	Bead beating and Wizard Genomic DNA Purification (Promega)	16S	Illumina Miseq	V4	QIIME (1.8)	Greengenes database
CeD/NCWS	Nobel et al.^59^	Stool	Frozen unprocessed within 24 hr of collection; −80°C	MagAttract PowerSoil Kit (Qiagen)	16S	Illumina Miseq	V3-V4	DADA2 (1.10.1) R (3.6.1)	Greengenes database
CeD/NCWS	Nylund et al.^60^	Stool	Collected by patient; −20°C followed by −70°C	Repeated beat beating with KingFisher InviMag Stool DNA kit	16S	Illumina MiSeq	V3-V4	DADA2 DECIPHER	Ribosomal Database Project (11.5)
NCWS	Dieterich et al.^47^	Stool	Stored at −20°C	QIAamp Fast DNA Stool Mini Kit (Qiagen)	16S	Illumina MiSeq	V3-V4	UPARSE pipeline METAGENassist	Ribosomal Database Project (11)
NCWS/FD	Shah et al.^64^	Biopsy (duodenum)	Collected with Brisbane Aseptic Biopsy device (MTW) into sterile RNAlater (Qiagen); −80°C	Repeat bead beating followed by automated extraction with Maxwell 16 Tissue DNA purification Kit (Promega)	16S	Illumina MiSeq	V6-V8	QIIME2 (V.2021.4) DADA2	SILVA database (138)
FD	Cervantes et al.^44^	Saliva Brush (gastric antrum & duodenum)	Saliva: collected prior to endoscopy Brush: Gently rubbed mucosa with a standard cytology brush (Olympus)	QIAamp ultraclean production Pathogen Mini Kit (Qiagen)	16S	Illumina MiSeq	V3-V4	Mothur	Ribosomal Database Project
FD	Fukui et al.^50^	Swab (oral) Brush (esophagus, gastric body, gastric antrum & duodenum)	Oral swab: Intraoral HydraFlock6 swab collected into sterile phosphate buffer saline; −80°C Brush: Under endoscopy, each location was brushed 10 times with a Cytology brush (Cook Medical) and collected into sterile phosphate buffer saline; −80°C	QuickGene DNA Tissue kit SII (KURABO)	16S	Illumina Miseq	V3-V4	Not described	Not described
FD	Igarashi et al.^53^	Aspirate (gastric)	A nasogastric tube was inserted into the stomach, and as much gastric fluid possible was aspirated using a disposable syringe	Ultra Clean Soil DNA Isolation Kit (Mo Bio Laboratories)	16S	Illumina Miseq	V3-V4	QIIME	TechnoSuruga Laboratory Microbial Identification Database DB-BA (10.0)
FD	Kim et al.^70^	Swab (oral) Brush (duodenum) Stool	Oral Swab: Swab from base of tongue by investigator; −20°C Brush: During endoscopy the second portion of the duodenum was brushed with a cytology brush (Olympus); −20°C Stool: Collected by patient into stabilization kit	QIAamp DNA Mini Kit (Qiagen)	16S	Ion Torrent Personal Genome Machine (Thermo Fisher Scientific)	V1-V2	QIME2	SILVA database
FD	Kovaleva et al.^71^	Stool	Collected by patient and then frozen; −70°C	Heated for 10 min each at 65 °C and 95 °C before homogenization. Samples were centrifuged, and DNA extraction was performed on supernatant using MagNA Pure Compact Nucleic Acid Isolation Kit I (Roche)	16S	Illumina Miseq	V3-V4	Trimmomatic (0.38) DADA2 (1.22) ALDEx2 package	SILVA database (138)
FD	Liu et al.^54^	Saliva	10 ml of saliva was collected in sterile 15-mL centrifuge tubes (Precidiag); −80°C	Performed externally at Exon Bio (Guangzhou, China)	16S	Not described	Not described	Not described	Not described
FD	Nakae et al.^56^	Aspirate (gastric)	Nasogastric tube was inserted into the stomach and as much fluid as possible was aspirated with a disposable syringe; −50°C	Ultra Clean Soil DNA Isolation Kit (Mo Bio Laboratories)	16S	T-RFLP	Not described	GeneScan (1.0) GeneMaths (1.0)	BLAST FASTA
FD	Shanahan et al.^65^	Biopsy (duodenum)	Collected with Brisbane Aseptic Biopsy device (MTW) into sterile RNAlater (Qiagen); −80°C	Repeat bead beating followed by automated extraction with Maxwell 16 Tissue DNA purification Kit (Promega)	16S	Illumina MiSeq	V6-V8	QIIME2 (V.2021.4) DADA2	SILVA database (138)
FD	Wauters et al.^68^	Biopsy (duodenum) Brush (duodenum)	Biopsy: Collected with Brisbane Aseptic Biopsy device (MTW) into sterile, nuclease-free tubes; −80°C Brush: Sterile brush (Zhuji Pengtian Medical Instrument Co.) used to collect sample from opposite biopsy collection location; −80°C	AllPrep DNA/RNA Mini kit (Qiagen)	16S	Illumina MiSeq	V4	LotuS DADA2 (1.6)	Ribosomal Database Project
FD	Zheng et al.^69^	Biopsy (duodenum) Brush (duodenum)	Biopsy: Collected with disposable sterile endoscopy clamp (Nanjing Medical Technology Co.); −80°C Brush: Collected with disposable sterile cell brush (Nanjing Medical Technology Co.); −80°C	Cetyltrimethylammonium Bromide (CTAB) method	16S	NovaSeq6000	Not described	FLASH (1.2.7) Mothur MUSCLE (3.8.31) QIIME (1.9.1)	SILVA database (138)

CeD, Celiac Disease; DADA2, Divisive Amplicon Denoising Amplicon 2; DNA, Deoxyribonucleic Acid; FD, Functional dyspepsia; NCGS, non-celiac gluten sensitivity; QIIME2, Quantitative Insights into Microbial Ecology 2; T-RFLP, Terminal Restriction Fragment Length Polymorphism.

Whilst two studies assessed the microbiota by shotgun metagenomic sequencing,^49,73^ most studies utilized 16S rRNA amplicon sequencing. 16S was performed on the Illumina Miseq platform in 70% of included articles; however, three studies utilized 454 pyrosequencing,^46,57,58^ and one employed terminal restriction fragment length polymorphism (TRFLP) methods.^56^ When sequencing by 16S, the variable regions V3 and V4 were most often amplified (19/30); however, three studies instead chose to amplify the V6-V8 regions^57,64,65^; two amplified V4-V6^46,52^; and one selected V1-V2.^70^ QIIME (14/30 studies) and DADA2 (9/30 studies) were the most commonly used pipelines for processing microbiota sequencing data; however, the analysis methods varied widely between studies. The database chosen to assign taxonomy was also highly variable; the Greengenes reference database was utilized in 9/30 studies; the Ribosomal Database Project database was used in 7/30 studies; and the SILVA database was employed in 6/30 articles.

### 3.3. The oral microbiota of CeD and FD patients has varied changes to diversity compared to controls

Seven included studies that assessed the oral microbiota by 16S sequencing, with four utilizing saliva samples,^44,54,62,67^ and the remaining studies employing oral cavity swabs.^50,52,70^ The oral microbiota was investigated in four studies of FD and three exploring CeD. CeD patients had a lower oral alpha diversity, as described by Iaffaldano et al.,^52^ Panelli et al.,^62^ and Tian et al.,^67^ Interestingly, Panelli et al.,^62^ only reported lower oral alpha diversity of refractory CeD patients compared to controls, but not aCeD or GFD CeD. Alternatively, in FD, no significant difference was identified in oral alpha diversity by Cervantes et al.,^44^ or Fukui et al.,^50^ or Kim et al.,^70^ although alpha diversity was not reported on by Liu et al.,^54^ (Table 3).

**Table 3.** Summary of findings: changes in microbiota diversity metrics.

Diagnosis	Study/Year	Specimen (location)	Alpha Diversity (test, p-value)	Beta Diversity (test, p-value)
CeD	Bodkhe et al.^43^	Stool Biopsy (duodenum)	**Stool**: Unchanged (Shannon, CeD vs DC, *p* = 0.55; CeD vs FDR, *p* = 0.48) **Duodenum**: Unchanged (Shannon, CeD vs DC, *p* = 0.78; CeD vs FDR, *p* = 0.68)	**Stool**: Unchanged (Bray-Curtis dissimilarity, ANOSIM, *p* = 0.058) **Duodenum**: Unchanged (Bray-Curtis dissimilarity, ANOSIM, *p* = 0.427)
CeD	Constante et al.^45^	Stool Biopsy (duodenum) Aspirate (duodenum)	Not described	**Duodenum**: Significantly different between CeD & controls (PERMANOVA on weighted Unifrac, *p* = 0.001)
CeD	D’Argenio et al.^46^	Biopsy (duodenum)	Unchanged (Faith’s Phylogenetic Diversity & observed species metric)	Significantly different between CeD & controls (unweighted Unifrac, ADONIS, *p* = 0.017; weighted Unifrac, ADONIS, *p* = 0.014)
CeD	Francavilla et al.^49^	Stool	Unchanged (richness, Simpson, Shannon)	Not described
CeD	Iaffaldano et al.^52^	Saliva Biopsy (duodenum)	**Saliva**: ↓ Diversity in aCeD compared to GFD CeD and controls (Chao1, Shannon, observed OTUs, phylogenetic metric)	**Saliva**: Significantly different between aCeD, GFD CeD and controls (unweighted Unifrac, ADONIS, *p* = 0.001; weighted Unifrac, ADONIS, *p* = 0.001)
CeD	Lopetuso et al.^55^	Stool	Not described for CeD compared to controls	Not described for CeD compared to controls
CeD	Nistal et al.^57^	Biopsy (duodenum)	↓Richness in GFD CeD compared to aCeD (*p* = 0.015)	Significantly different between aCeD, GFD CeD & controls (UniFrac distances, *p* ≤ 0.03)
CeD	Nistal et al.^58^	Biopsy (duodenum)	NS↓ diversity in CeD compared to controls (Chao 1, Shannon)	Unchanged (unweighted & weighted Unifrac, Morosita-Horn index)
CeD	Palmieri et al.^61^	Stool	Unchanged (Shannon, *p* = 0.07; Pielou’s, *p* = 0.75; observed features, *p* = 0.09; Faith’s phylogenetic diversity, *p* = 0.34)	Significantly different between CeD and controls (Jaccard similarity, *p* = 0.004; Bray-Curtis, *p* = 0.005; Unweighted UniFrac, *p* = 0.06; Weighted UniFrac, *p* = 0.001)
CeD	Panelli et al.^62^	Stool Saliva Biopsy (duodenum)	**Duodenum**: ↓ diversity in CeD and pCeD compared to FD (Richness, Shannon, *p* < 0.05) ↓ diversity in aCeD and pCeD compared to FD (Chao1, *p* < 0.05) **Saliva**: ↓ diversity in rCeD compared to FD, GFD CeD and aCeD (Richness, Chao1, *p* < 0.05) **Stool**: ↑ diversity in GFD CeD and aCeD compared to FD (Shannon, *p* < 0.05)	**Duodenum**: Significantly different between all groups (PERMANOVA on UniFrac distances, *p* < 0.05) **Saliva**: Unchanged **Stool**: Unchanged
CeD	Rawson et al.^72^	Stool	Unchanged (Shannon, OTU abundance)	Unchanged (Bray-Curtis)
CeD	Serena et al.^63^	Stool	↑ diversity in aCeD compared to GFD CeD and controls (Faith’s phylogenetic diversity, *p* < 0.05)	Significantly different between aCeD and both GFD CeD and controls (unweighted UniFrac distances, *p* < 0.05)
CeD	Shi et al.^66^	Stool	↓ diversity in CeD compared to controls (observed species, *p* = 0.041; Chao1, *p* = 0.042; ACE, *p* = 0.040)	Significantly between CeD and controls (ANOSIM on UniFrac distances, *p* = 0.0005)
CeD	Slager et al.^73^	Stool	Unchanged between GFD CeD compared to controls (Shannon)	Significantly different between GFD CeD and controls (Bray-Curtis, *p* < 0.05)
CeD	Tian et al.^67^	Saliva	↓ diversity in GFD CeD compared to healthy controls (Shannon, *p* = 0.016)	Not described
CeD/NCWS	Garcia-Mazcorro et al.^51^	Stool Biopsy (duodenum)	**Duodenum**: ↓diversity in CeD (Shannon, *p* = 0.02) Unchanged by other metrics (richness, *p* = 0.8; PD whole tree, *p* = 0.8; Chao1, *p* = 0.9) **Stool**: Unchanged (Shannon, richness, PD whole tree, Chao1)	**Duodenum**: Unchanged (weighted and unweighted UniFrac distances)
CeD/NCWS	Nobel et al.^59^	Stool	Unchanged between all groups (Shannon)	Significantly different between NCGS, CeD and controls (Weighted UniFrac, *p* < 0.05; Bray Curtis, *p* < 0.001)
CeD/NCWS	Nylund et al.^60^	Stool	Unchanged between all groups (Chao1, diversity)	Unchanged (Bray-Curtis dissimilarity)
NCWS	Dieterich et al.^47^	Stool	Not described	Not described
NCWS/FD	Shah et al.^64^	Biopsy (duodenum)	↑ diversity in total FD patient cohort compared to controls at species level (Shannon, *p* = 0.039; Simpson, *p* = 0.044) Unchanged at genus level (Shannon, *p* = 0.14; Simpson) Unchanged between FD with NCWS and controls at genus and species level (Shannon, *p* = 0.37 and *p* = 0.24, respectively)	Unchanged (weighted UniFrac, ADONIS, *p* = 0.21; unweighted UniFrac, ADONIS, *p* = 0.11; Bray Curtis, ADONIS, *p* = 0.33)
FD	Cervantes et al.^44^	Saliva Brush (gastric antrum & duodenum)	**Saliva**: Unchanged (Shannon, *p* = 0.08) **Stomach**: Unchanged (Shannon, *p* = 0.82) **Duodenum**: Unchanged (Shannon, *p* = 0.40)	**Saliva**: Significantly different between dyspepsia and achalasia controls (PCoA, AMOVA, *p* = 0.005) **Stomach**: Unchanged (PCoA, AMOVA, *p* = 0.82) **Duodenum**: Unchanged (PCoA, AMOVA, *p* = 0.40)
FD	Fukui et al.^50^	Swab (oral) Brush (esophagus, gastric body, gastric antrum & duodenum)	Unchanged (observed species, Chao1, Shannon)	Significantly different between FD and controls (unweighted Unifrac, PERMANOVA, *p* = 0.0001; weighted Unifrac, PERMANOVA, *p* = 0.0001)
FD	Igarashi et al.^53^	Aspirate (gastric)	Unchanged (Chao1)	Significantly different between FD and controls (Euclidian distance, ANOSIM, *p* < 0.01)
FD	Kim et al.^70^	Swab (oral) Brush (duodenum) Stool	**Oral**: Unchanged (Chao1, *p* = 0.46) **Duodenum**: Unchanged (Chao1, *p* = 0.79) **Stool**: Unchanged (Chao1, *p* = 0.31)	**Oral**: Unchanged (UniFrac, *p* = 0.68) **Duodenum**: Unchanged (UniFrac, *p* = 0.81) **Stool**: Unchanged (UniFrac, *p* = 0.11)
FD	Kovaleva et al.^71^	Stool	Unchanged (Chao1, *p* = 1.0; Shannon, *p* = 0.6; ACE index, *p* = 0.9)	Not described
FD	Liu et al.^54^	Saliva	Not described	Different between FD and controls (Bray-Curtis distances)
FD	Nakae et al.^56^	Aspirate (gastric)	Not described	Significantly different between FD and controls (Bray-Curtis, *p* < 0.001)
FD	Shanahan et al.^65^	Biopsy (duodenum)	Unchanged (Shannon, *p* = 0.3; Chao1, *p* = 0.08)	Unchanged (Weighted UniFrac, ADONIS, *p* = 0.17)
FD	Wauters et al.^68^	Biopsy (duodenum) Brush (duodenum)	**Brush**: ↓ Richness in FD compared to controls, when not on PPIs (*p* = 0.03) **Biopsy**: Unchanged (Richness, Shannon, Simpson)	**Brush**: Significantly different between FD on or off PPIs and controls (ADONIS, *p* < 0.001) **Biopsy**: Significantly different between FD on or off PPIs and controls (ADONIS, *p* = 0.03)
FD	Zheng et al.^69^	Biopsy (duodenum) Brush (duodenum)	↑ Diversity in FD patients compared to controls (ACE, *p* = 0.0003; Shannon, *p* = 0.0236; observed species, *p* = 0.0008)	Significantly different between FD and controls (AMOVA, *p* = 0.021)

ANOSIM, Analysis of similarities; CeD, Celiac Disease; DC, Disease controls; FDR, First-degree relative; GFD, Gluten free diet; NCGS, non-celiac gluten sensitivity; NS, not significant; OTU, operational taxonomic units; PERMANOVA, Permutational Multivariate Analysis of Variance; ↑, increased, ↓, decreased.

The beta diversity of the oral microbiota was reported to be significantly altered in CeD patients by Iaffaldano et al.,^52^ However, Panelli et al.,^62^ did not identify a change in CeD beta diversity, and Tian et al.,^67^ did not describe beta diversity. In FD patients, three of the included studies reported a difference to oral beta diversity when compared to controls;^44,50,54^ however, Kim et al.,^70^ found no significant change (Table 3).

A variety of differences in microbial abundance were described for the oral microbiota of both FD and CeD patients (Supplementary Table S1). Tian et al.,^67^ described differences in the CeD oral microbiota at a species level which included lower *Actinomyces* spp. and higher *Lactobacillus* spp. and *Leptotrichia* spp. Meanwhile, Iaffaldano et al.,^52^ reported reduced Bacteroidetes and Firmicutes and increased Proteobacteria in CeD patients compared to controls. Furthermore, they reported reduced *Leptotrichia*, *Prevotella*, and *Streptococcus* genera, as well as increased Neisseria in CeD patients.^52^ Similarly, Panelli et al.,^62^ reported that *Neisseria* was elevated in CeD when compared to an FD control cohort, whilst *Acinetobacter* genus was reduced. Interestingly, Kim et al.,^70^ found that FD patients had significantly higher oral *Neisseria* compared to healthy controls. FD patients were described by Cervantes et al.,^44^ to more of the genera *Anoxybacter*, *Cohnella*, and *Veilonella*, and less *Parvimonas* and *Peptostreptococcus* when compared to achalasia controls. Within saliva, Liu et al.,^54^ described higher *Kingella* and *Abiotrophia* genera in FD compared to controls. Fukui et al.,^50^ assessed the microbiota across numerous gastrointestinal sites and found that the phylum Firmicutes and genus *Streptococcus* were elevated at all sites, including the oral compartment, in FD patients when compared to controls.

### 3.4. The gastric microbiota of FD patients has specific microbial alterations but no change in alpha diversity compared to controls

Four studies compared the gastric microbiota of FD patients with controls. Cervantes et al.,^44^ and Fukui et al.,^50^ assessed the microbiota in gastric brush samples, whilst Igarashi et al.,^53^ and Nakae et al.,^56^ utilized gastric aspirate. No differences in gastric alpha diversity were identified in any of these studies. Nakae et al.,^56^ Igarashi et al.,^53^ and Fukui et al.,^50^ described altered beta diversity in the gastric microbiota of FD patients, whereas Cervantes et al.,^44^ did not identify any differences in beta diversity of FD compared to controls within the stomach (Table 3).

Cervantes et al.,^44^ described the gastric microbiota of dyspepsia patients to be characterized by lower *Bifidobacterium*, *Clostridium*, and *Lactobacillus* genera. According to Igarashi et al.,^53^ the genus *Bacteroides* was higher in FD patients, whereas *Edaphobacter* was lower in the gastric fluid. Igarashi et al., also reported higher *Bifidobacterium longum* species in the FD gastric microbiota. Nakae et al.,^56^ instead reported lower gastric *Prevotella* and higher *Bifidobacterium* in FD compared to controls. Fukui et al.,^50^ described elevated Firmicutes and *Streptococcus* in FD patients (Supplementary Table S2).

### 3.5. Studies on duodenal microbiota in FD, CeD, and NCGS patients revealed diverse impacts on microbial abundance and diversity

The duodenal microbiota was examined in seven studies assessing FD, six exploring CeD, and two investigating NCGS. The duodenal microbiota was analyzed from biopsy samples in most studies; however, Cervantes et al.,^44^ Fukui et al.,^50^ and Kim et al.,^70^ utilized duodenal brush samples, whilst Wauters et al.,^68^ and Zheng et al.,^69^ employed both brush and biopsy samples to assess the duodenal microbiota. There was little consensus on how these conditions impacted alpha diversity within the duodenum. Both Panelli et al.,^62^ and Garcia-Mazcorro et al.,^51^ described lower alpha diversity in CeD. However, the remaining four studies assessing CeD microbiota did not identify a significant difference in alpha diversity within the duodenum.^43,46,57,58^ Similarly, Shah et al.,^64^ and Garcia-Mazcorro et al.,^51^ did not find any alteration to alpha diversity in NCGS patients. In FD patients, Zheng et al.,^69^ and Shah et al.,^64^ described higher alpha diversity whilst Wauters et al.,^68^ reported lower alpha diversity. Cervantes et al.,^44^ Shanahan et al.,^65^ Kim et al.,^70^ and Fukui et al.,^50^ did not report any differences in FD alpha diversity within the duodenum (Table 3).

With respect to duodenal beta diversity, D’Argenio et al.,^46^ Nistal et al.,^57^ and Panelli et al.,^62^ described differences in CeD patients compared to controls, whereas the remaining studies on CeD did not.^43,45,51,58^ No differences in beta diversity were reported in NCGS when comparing patients to controls.^51,64^ Similarly, Shah et al.,^64^ Cervantes et al.,^44^ Kim et al.,^70^ and Shanahan et al.,^65^ did not describe a difference in FD duodenal beta diversity compared to controls. However, significant differences in beta diversity between FD patients and controls were described by Wauters et al.,^68^ Zheng et al.,^69^ and Fukui et al.^50^ (Table 3).

Differences in bacterial abundance were described in the 14 studies which assessed the duodenal microbiota (Supplementary Table S3). Bodkhe et al.,^43^ identified ASVs belonging to many genera which were differentially abundant between groups; including elevated *Lactobacillus*, *Megasphaera*, and *Helicobacter*; and reduced *Actinomyces*, *Streptococcus*, *Gemella*, and *Bifidobacterium* in CeD compared to healthy FDRs. Constante et al.,^45^ investigated the microbiota at different sites within the duodenum and found that the abundance of *Escherichia coli* in the superior duodenum (D1), *Prevotella salivae* in the descending duodenum (D2), and *Neisseria* in the inferior duodenum (D3) was elevated in CeD compared to disease controls. Alternatively, the genus *Staphylococcus* in D2 and the species *Staphylococcus epidermidis* in D1 and D3 were reduced in CeD patients compared to controls. Interestingly, D’Argenio et al.,^46^ also described increased *Neisseria* genus and the family Neisseriaceae in active CeD patients compared to controls. In a 2012 study, Nistal et al.,^57^ described significantly lower *Streptococcus* abundance in the duodenum of CeD. However, in a 2016 study by Nistal et al.,^58^ *Streptococcus* and *Lactobacillus* were not significantly altered in CeD patients. The genus *Neisseria* was also higher in the duodenum of aCeD patients as reported by Panelli et al.,^62^ when compared to an FD control cohort. Furthermore, they described lower Rothia in aCeD and reduced *Streptococcus* in both aCeD & GFD CeD compared to the FD control cohort.

Garcia-Mazcorro et al.,^51^ explored the microbiota of CeD and NCGS and found that in CeD patients, *Novispirillum* and *Brevundimonas* were higher, whereas the genera *Actinobacillus* and *Finegoldia* were increased in NCGS patients compared to controls. Shah et al.,^64^ instead found that the NCGS microbiota was characterized by elevated *Streptococcus*, *Faecalibaculum*, and *Actinomyces* compared to FD patients without wheat sensitivity. Furthermore, when comparing FD patients to controls, Shah et al.,^64^ noted that elevated *Neisseria* and *Prevotella*, and reduced *Streptococcus* were representative of FD. Shanahan et al.,^65^ reported similar trends to Shah et al., in FD patients; however, it should be noted that their cohorts overlap partially. Shanahan et al.,^65^ additionally described lower *Rothia* genus in FD patients when compared to controls. Alternatively, Cervantes et al.,^44^ reported that *Rothia*, along with *Haemophilus* and *Eubacterium*, was elevated in dyspepsia patients. Wauters et al.,^68^ described reduced genera *Neisseria* and *Porphyromonas* in the duodenum of FD, Whilst Zheng et al.,^69^ outlined a variety of changes in the duodenal microbiota including increased *Alloprevotella*, *Peptostreptococcus*, *Sutterella*, and *Faecalibacterium*, as well as reduced *Catonella* in FD patients. Kim et al.,^70^ reported significantly higher *Streptococcus* in the duodenum of FD patients, which is consistent with the findings of Fukui et al.,^50^ who observed elevated *Streptococcus* across all gastrointestinal sites in FD patients, including the duodenum. Additionally, Fukui et al.,^50^ found that the phylum *Firmicutes* was elevated in the duodenum.

### 3.6. Faecal microbiota in FD, CeD, and NCGS patients showed mixed alpha and beta diversity, and varied changes to microbial taxa

The fecal microbiota was evaluated in 16 studies, two of which utilized shotgun metagenomic sequencing,^49,73^ as opposed to 16S sequencing. Ten studies focussed on CeD; three examined both CeD and NCGS; two studies explored FD; and one study addressed only NCGS. Most studies reported that alpha diversity within the fecal microbiota was similar between cases and controls.^43,49,51,59–61,70–73^ However, Panelli et al.,^62^ and Serena et al.,^63^ observed higher alpha diversity in CeD patients, whilst Shi et al.,^66^ noted lower alpha diversity in the stool of CeD patients (Table 3). The findings on beta diversity within the stool were highly heterogenous. Five studies on CeD reported significant differences in beta diversity between cases and controls,^59,61,63,66,73^ whereas three other studies found no such differences.^43,60,62,72^ Similarly, in NCGS, Nobel et al.,^59^ observed a difference in beta diversity between cases and controls whilst Nylund et al.,^60^ did not detect any differences. The fecal beta diversity in FD patients was similar to controls in the study by Kim et al.,^70^ and was not investigated in the article by Kovaleva et al.^71^

Across the different studies, the specific microbes which were reportedly altered in the stools of FD, CeD, and NCGS varied widely (Supplementary Table S4). In the Bodkhe et al.,^43^ study of CeD they described higher abundance of ASVs from the genera *Akkermansia*, *Dorea*, *Lactobacillus*, and *Prevotella* compared to disease controls. Alternatively, Constante et al.,^45^ reported that *Prevotella*, as well as *Bacteroides*, was higher in the feces of CeD patients compared to controls. Lopetuso et al.,^55^ explored the CeD stool microbiota amongst a larger disease cohort and identified significant associations between CeD and the genera *Paraprevotella*, *Anaerofustis*, *Pseudoramibacter*, and *Eubacterium*. Rawson et al.,^72^ reported reduced genus *Methanobrevibacter* in GFD CeD when compared to healthy controls and controls also consuming GFD. According to Serena et al.,^63^ the CeD, fecal microbiota was characterized by higher Bacteroidetes phylum and lower Firmicutes, when compared to controls. Shi et al.,^66^ however reported that the phylum Proteobacteria was positively associated with the CeD fecal microbiota. Shi et al.,^66^ also described increased *Streptococcus*, *Lactobacillus*, and *Veillonella* genera and reduced *Blautia*.

Through shotgun metagenomic sequencing, Slager et al.,^73^ identified differences in the fecal microbiota of CeD patients at a species level, including higher *Bacteroides* spp., *Clostridium* spp. CAG:253, *Roseburia hominis*, *Roseburia inulinivorans*, and *Haemophilus parainfluenzae*. Francavilla et al.,^49^ similarly employed shotgun sequencing to assess the CeD microbiota and also reported elevated *Roseburia inulinivorans* and *Haemophilus parainfluenzae*. Furthermore, Francavilla et al.,^49^ found that the abundance of *Streptococcus sanguinis* and *Veillonella atypica* was higher in treated CeD patients, whilst that of *Bifidobacterium longum* was lower. Interestingly, Palmieri et al.,^61^ also reported lower *Bifidobacterium longum* to be associated with CeD, as well as higher Bacteroides abundance. Conversely, Panelli et al.,^62^ reported that *Bifidobacterium longum* was elevated in active CeD patients compared to an FD control cohort. Furthermore, Panelli et al.,^62^ described higher *Blautia* and *Dorea* genera in CeD.

Garcia-Mazcorro et al.,^51^ assessed the fecal microbiota in both CeD and NCGS, finding that both conditions were associated with the increased Ruminococcaceae family. Additionally, CeD patients had an increased abundance of Mogibacteriaceae, whilst NCGS patients showed higher levels of Oxalobacteraceae within their feces. Nobel et al.,^59^ similarly investigated both CeD and NCGS and found that *Ruminococcus* and *Turicibacter* were elevated in both of these conditions when compared to controls. Furthermore, Nobel et al.,^59^ reported that *Blautia* was reduced in NCGS, whilst *Bifidobacterium* was reduced in CeD. Nylund et al.,^60^ did not identify any significant differences in microbial abundance in the stool of CeD or NCGS patients; however, they did observe non-significantly lower *Bifidobacterium* in CeD and NCGS compared to controls. Dieterich et al.,^47^ assessed patients with NCGS and reported that the Ruminococcaceae and Peptostreptococcaceae families were elevated, whereas the Porphyromonadaceae family was reduced in stool when compared to controls.

The fecal microbiota of FD patients was reported by Kim et al.,^70^ to contain significantly lower *Butyricicoccus* and *Faecalibacterium*, whereas Kovaleva et al.,^71^ described increased *Holdemanella*, *Agathobacter*, *Lactococcus*, and *Stenotrophomonas*; and a reduced *Frisingicoccus*, *Hungatella*, *Eisenbergiella*, *Parabacteroides*, *Bilophila*, and *Flavonifractor* in FD patients with overlapping irritable bowel syndrome, when compared to controls. Thus, there was little consistency in the differences in specific bacterial taxa between studies.

### 3.7. Studies reporting on the functional capacity of the gastrointestinal microbiome highlight changes to metabolic pathways and gene functions

Nine of the included studies described differences in the functional capacity of the gastrointestinal microbiome compared to controls (Table 4). In the saliva of aCeD patients, Iaffaldano et al.,^52^ reported that there was higher potential for degrading amino acids, lipids, and ketone bodies and fewer genes associated with polysaccharide metabolism, when compared to both GFD CeD and controls. In CeD patients, Bodkhe et al.,^43^ predicted lower Xaa-pro dipeptidase within stool, but not the duodenum. Importantly, Xaa-pro dipeptidase has the capacity to hydrolyze specific dipeptide sequences found within gluten. Constante et al.,^45^ identified differences in the CeD duodenum including reduced glutamate carboxypeptidase genes and increased serine proteases, as predicted through PICRUSt2 analysis. Within the stool, Palmieri et al.,^61^ described higher potential for tetrapyrrole biosynthesis in the CeD microbiota. Francavilla et al.,^49^ assessed microbial function in the stool of CeD by metagenomic sequencing and found fewer genes associated with biosynthesis of starch, 4-hydroxybenzoate, and amino acids, as well as more microbial genes for nitrate reduction and L-rhamnose degradation. Also, utilizing shotgun metagenomic sequencing, Slager et al.,^73^ likewise reported more genes involved in the rhamnose degradation pathway, as well as those associated with the mannitol cycle, purine nucleotide degradation and sugar biosynthesis. Furthermore, Slager et al.,^73^ described a reduction in genes related to anaerobic energy metabolism and gluconeogenesis in CeD patients, compared to controls. Interestingly, Garcia-Mazcorro et al.,^51^ did not identify any significant differences in predicted microbial function in the stool or duodenum of CeD and NCGS patients.

**Table 4.** Summary of findings: changes in microbial function.

Diagnosis	Study/Year	Specimen (location)	Functional microbial changes
CeD	Bodkhe et al.^43^	Stool Biopsy (duodenum)	**Stool**: ↓ KO abundance for Xaa-pro dipeptidase (K01271) inferred from predicted metagenome (ANOVA, *p* = 0.044) in CeD compared to controls
CeD	Constante et al.^45^	Stool Biopsy (duodenum) Aspirate (duodenum)	**Duodenum**: ↓glutamate carboxypeptidase genes in D2 of CeD (predicted with PICRUSt2) ↑ serine protease in D1, D2, & D3 of CeD (predicted with PICRUSt2)
CeD	Francavilla et al.^49^	Stool	↓ microbial genes involved in the 4-hydroxybenzoate biosynthesis, starch biosynthesis, *Bifidobacterium* shunt, and amino acids biosynthesis (L-arginine biosynthesis I, II, and IV) in CeD compared to controls ↑ microbial genes involved in nitrate reduction and L-rhamnose degradation in CeD compared to controls
CeD	Iaffaldano et al.^52^	Saliva Biopsy (duodenum)	**Saliva**: ↑ potential for degradation of amino acids, metabolism of lipid and ketone bodies, and microbial antioxidant defense mechanisms in aCeD compared to GFD CeD and controls ↓ genes associated with polysaccharide metabolism in aCeD compared to GFD CeD and controls
CeD	Palmieri et al.^61^	Stool	↓ tetrapyrrole biosynthesis I from glutamate and tetrapyrrole biosynthesis I from glycine in CeD compared to controls (PICRUSt)
CeD	Slager et al.^73^	Stool	↓ microbial genes related to transmembrane transport and RadC-like JAB domain ↑ genes involved in the rhamnose degradation pathway, mannitol cycle, NAD/NADH phosphorylation, purine nucleotide degradation, dTDP-N-acetylviosamine biosynthesis, and dTDP-3-acetamido-3,6-dideoxy-a-D-galactose biosynthesis (MetaCyc pathways profiling with HUMAnN3) ↓ microbial genes associated with anaerobic energy metabolism and gluconeogenesis III (MetaCyc pathways profiling with HUMAnN3)
CeD/NCWS	Garcia-Mazcorro et al.^51^	Stool Biopsy (duodenum)	**Duodenum**: No significant changes NS↓ Flavonoid biosynthesis, riboflavin metabolism, and ↑ dioxin degradation in CeD compared to controls **Stool**: No significant changes
FD	Cervantes et al.^44^	Saliva Brush (gastric antrum & duodenum)	**Saliva**: ↑ pathways for purine, cysteine-methionine, thiamine, and nitrogen metabolism; biosynthesis of ubiquinone, lipopolyshaccaride, tRNA, and terpenoid; and amino acid related enzymes predicted in dyspepsia compared to achalasia compared to controls (PICRUSt) ↓ pathways for metabolism of various saccharides, as well as proteins involved in bacterial cell motility and chemotaxis predicted in dyspepsia compared to achalasia compared to controls (PICRUSt) **Stomach**: ↑ pathways involved in tricarboxylic acid cycle, peptidases, protein folding and associated processing and ↓ transporters, and proteins involved in replication, recombination and repair predicted in dyspepsia compared to achalasia compared to controls (PICRUSt) **Duodenum**: No significant changes
FD	Zheng et al.^69^	Biopsy (duodenum) Brush (duodenum)	Predicted ureolysis and fumarate respiration functions were significantly different between FD and controls (FAPROTAX, *p* < 0.05)

ANOVA, Analysis of variance; CeD, Celiac Disease; DC, Disease controls; FD, Functional dyspepsia; FDR, First-degree relative; GFD, Gluten free diet; NCGS, non-celiac gluten sensitivity; OTU, operational taxonomic units; PICRUSt2, Phylogenetic Investigation of Communities by Reconstruction of Unobserved States.

In the duodenum of FD patients, Zheng et al.,^69^ found that predicted ureolysis and fumarate respiration were altered compared to controls. Cervantes et al.,^44^ however, did not identify any significant differences in the duodenal microbiota function of dyspepsia patients compared to an achalasia control group. Nonetheless, in the saliva, Cervantes et al.,^44^ reported that pathways for purine, cysteine-methionine, thiamine, and nitrogen metabolism and biosynthesis of ubiquinone, lipopolysaccharide, tRNA, and terpenoid were predicted to be increased in dyspepsia. Furthermore, there was a reduced predicted capacity to metabolize various saccharides. Cervantes et al., also described elevated pathways involved in the tricarboxylic acid cycle, peptidases, protein folding and associated processing and reduced transporters, and proteins involved in replication, recombination, and repair, within the stomach of dyspepsia patients.

### 3.8. Re-analysis of published datasets reveals changes to predicted gluten digesting genes

After assessing data availability statements and contacting authors, 5/14 articles had available 16S sequence data from the duodenal microbiota of CeD, NCGS, or FD and corresponding controls, with sufficient metadata to support re-analysis.^43,45,51,64,65^ These datasets were highly heterogeneous with Constante et al.,^45^ utilizing V3 primers; Bodkhe et al.,^43^ and Garcia-Mazcorro et al.,^51^ amplifying the V4 region; and Shah et al^64^., and Shanahan et al^65^., using primers for the V6-V8 variable region. Due to the different primer selections and sequencing batch effects, raw data from these articles was analyzed separately with a consistent pipeline. The exception to this was the sequence data from the two Australian studies by Shah et al.,^64^ and Shanahan et al.,^65^ as these cohorts overlapped and were sequenced together; thus, they have been combined into one dataset and analyzed as such.

Assessing the alpha diversity using the Shannon index (Table 5), we observed no significant differences between CeD patients and controls in data derived from Bodkhe et al. (*p* > 0.99). There was higher alpha diversity (*p* = 0.03) in CeD patients from Constante et al. Conversely, CeD patients from the study by Garcia-Mazcorro et al., had lower alpha diversity (*p* = 0.02) but no difference for NCGS patients (*p* = 0.98). Finally, the alpha diversity of FD patients was significantly reduced (*p* = 0.0008) in the Shah et al., and Shanahan et al., dataset. To evaluate the richness of the microbiota, the Chao1 index was employed and no significant differences in richness were identified in the datasets from Bodkhe et al., (*p* > 0.99), Constante et al., (*p* = 0.78), and Garcia-Mazcorro et al., (CeD, *p* = 0.64; NCGS, *p* = 0.84) when compared to controls. There was, however, significantly reduced richness in FD patients from the Shah et al., and Shanahan et al., dataset (*p* = 0.0015). The Bray-Curtis beta diversity identified significant differences between the cases and controls in data from Constante et al., (PERMANOVA, *p* = 0.041); Garcia-Mazcorro et al., (PERMANOVA, *p* = 0.018); and the Shah et al., and Shanahan et al., dataset (PERMANOVA, *p* = 0.001). However, there were no significant differences in beta diversity in the Bodkhe et al., dataset (PERMANOVA, *p* = 0.594).

**Table 5.** Summary diversity metrics and random forest analysis calculated on raw sequence data analyzed through a uniform bioinformatic pipeline.

				Alpha Diversity (Shannon index)			Alpha Diversity (Chao1 index)			Beta diversity (PERMANOVA on Bray-Curtis dissimilarity	ROC Curve of Random Forest Classifier
Study/Year	Location	Case (n)	Control (n)	CeD	NCGS	FD	CeD	NCGS	FD	P value	Area under curve
Bodkhe et al.^43^	India	aCeD (23)	Healthy FDR (15) DC (24)*	- (*p* > 0.99)			- (*p* > 0.99)			0.594	0.54
Constante et al.^45^	Canada	aCeD (24)	Healthy (30) Hospital controls (11)	↑ (*p* = 0.03)*			- (*p* = 0.78)			0.041*	0.6
Garcia-Mazcorro et al.^51^	Mexico	aCeD (6) aNCGS (12)	Healthy (12)	↓ (*p* = 0.02)*	- (*p* = 0.98)		- (*p* = 0.64)	- (*p* = 0.84)		0.018*	CeD: 0.65 NCGS: 0.61
Shanahan et al.^65^ Shah et al.^64^	Australia	FD (61)	Hospital controls (38)			↓ (*p* = 0.0008)*			↓ (*p* = 0.0015)*	0.001*	0.56

*p < 0.05; ↑, increased; ↓, decreased.

**Figure 1.**
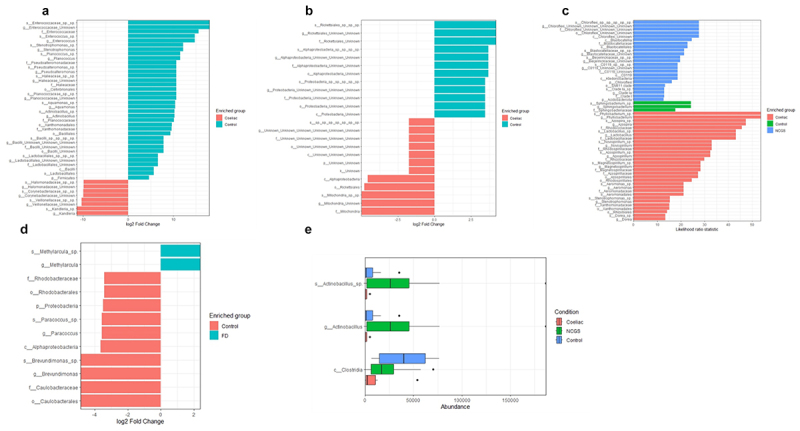
Differentially abundant taxa within the duodenal microbiota of patients with functional dyspepsia, celiac disease or non-celiac gluten sensitivity. Raw data from published microbiota sequencing datasets was analyzed with a consistent pipeline and the differentially abundant taxa identified by EdgeR analysis are presented for (a) Bodkhe et al. (b) Constante et al. (c) Garcia-Mazcorro et al. and (d) Shah et al. and Shanahan et al. (e) LEfSe results from Garcia-Mazcorro et al. *p* < 0.05.

To attempt to identify a consensus in taxa that may drive changes between cases and controls, we analyzed differential abundance with ANCOM-BC2, LEfSe, and EdgeR. ANCOM-BC2 did not identify markers in any of the datasets, whilst LEfSe analysis with a cutoff of 0.05, revealed only one study from which markers were identified. Within the Garcia-Mazcorro et al., dataset, *Actinobacillus* (*p* = 0.037) was elevated in NCGS patients, and Clostridia was enriched in controls (*p* = 0.025) (Figure 1(e)). Meanwhile, EdgeR differential analysis identified significantly altered microbes between cases and controls in each of the included datasets. Forty-four differentially abundant taxa were identified in CeD by Bodkhe et al. (Figure 1a). This included an increase in taxa belonging to Enterococcaceae, *Stenotrophomonas*, *Planococcus*, *Actinobacillus*, and reduced Corynebacteriaceae and Veillonellaceae. Within the dataset from Constante et al., 24 markers of differential abundance were detected in CeD including reduced abundance of taxa belonging to *Rickettsiales*, *Alphaproteobacteria*, *Proteobacteria*, and an increase in several uncultured microbes (Figure 1b). A total of 57 taxa were differentially abundant in CeD, NCGS, and controls from Garcia-Mazcorro et al. (Figure 1c). Controls were characterized by an abundance of *Sphingobacterium*, whilst CeD had a greater abundance of *Phyllobacterium*, *Lactobacillus*, *Azospira*, *Novispirillum*, and *Dorea*. NCGS had an abundance of species belonging to Chloroflexi and an increase in genera belonging to *Blastocatellaceae* and *Beijerinckiaceae*. Finally, there was only 12 markers which differentiated FD patients from controls in the Shah et al., and Shanahan et al., dataset (Figure 1d). FD patients had elevated *Methylarcula* and reduced *Proteobacteria* phylum and *Paracoccus* and *Brevundimonas* genera. Despite analyzing the datasets with a consistent pipeline, no clear signal in differentially abundant taxa is evident across the studies included in the re-analysis. To further investigate if the microbiota could be used to distinguish cases from controls, we performed supervised machine learning with random forest classifiers for each study. ROC curves were generated with the outputs to look at the performance of the machine learning methods to separate groups and across the board it was found that the area under the curve was approaching 0.5; thus, there was no discrimination, and the microbiota was a poor predictor of disease state (Table 5).

**Figure 2.**
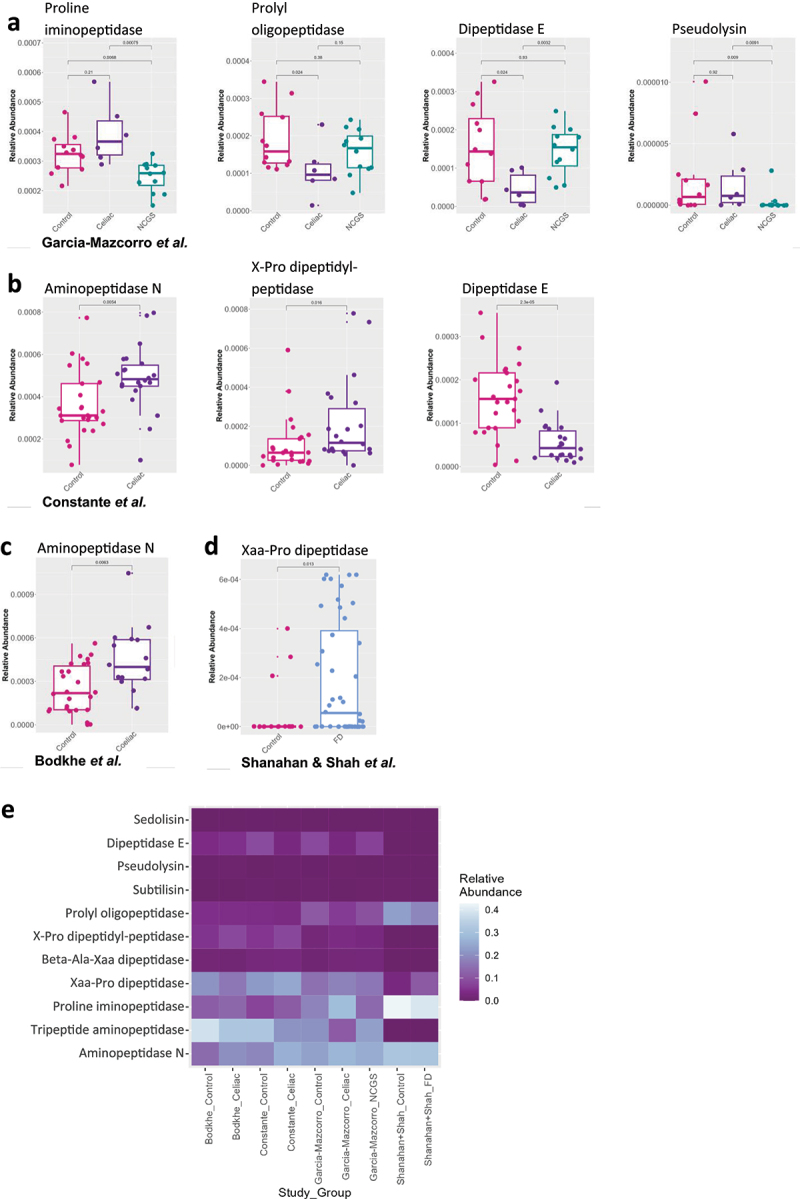
Significantly altered microbial genes with capacity to aid in gluten digestion. Functional capability of duodenal microbiota in published datasets was predicted with phylogenetic investigation of communities by reconstruction of unobserved states 2 (PICRUSt2). The relative abundance of genes reported to be involved in the breakdown of gluten were compared between cases and controls for each dataset. Significantly altered pathways (*p* < 0.05) are shown here for (a) Garcia-Mazcorro et al. (b) Constante et al., (c) Bodkhe et al., and (d) Shanahan et al., and Shah et al. (e) Heatmap showing average relative abundance of each gene assessed for all datasets included.

Next, we assessed if the functional capacity of the microbiome was altered in patients with CeD, NCGS, or FD, in a way which would impact the digestion of gluten. To investigate this, the function of the duodenal microbiota from the published datasets was predicted with PICRUSt2. The relative abundance of genes reported to have the capacity to breakdown gluten were compared between cases and controls for each dataset (Figure 2e). No significant differences in the abundance of sedolisin (K05998), subtilisin (K01342), beta-Ala-Xaa dipeptidase (K01274), or tripeptide aminopeptidase (K01258) were identified for any of the included datasets (Figure 2e). The predicted relative abundance of the aminopeptidase N gene (K01256) was significantly higher in the CeD cohorts from both Bodkhe et al., (*p* = 0.0063, Figure 2c) and Constante et al., (*p* = 0.0054, Figure 2b) when compared to their respective controls. Furthermore, within the dataset from Constante et al., the relative abundance of X-Pro dipeptidyl-peptidase (K01281) was increased in CeD patients (*p* = 0.016) and the abundance of dipeptidase E (K05995) was reduced (*p* = 0.000023) in CeD, compared to controls (Figure 2b). Dipeptidase E relative abundance was also decreased compared to controls (*p* = 0.024) and NCGS (*p* = 0.00032), in CeD patients from the Garcia-Mazcorro et al., dataset (Figure 2a). Furthermore, CeD patients from the Garcia-Mazcorro et al., cohort had reduced abundance of prolyl oligopeptidase (K01322) compared to controls (*p* = 0.024, Figure 2a). The NCGS patients within the Garcia-Mazcorro et al., dataset displayed significantly lower predicted proline iminopeptidase (K01259) relative abundance compared to controls (*p* = 0.0068) and CeD patients (*p* = 0.00075). NCGS patients additionally had lower pseudolysin (K01399) abundance compared to both controls (*p* = 0.009) and CeD patients (*p* = 0.0091, Figure 2a). Finally, in the FD cohorts from Shanahan et al., and Shah et al., there was significantly higher predicted relative abundance of Xaa-Pro dipeptidase (K01271) when compared to controls (*p* = 0.013, Figure 2d). These data suggest that patients with GRDs may have an altered microbial capacity to digest gluten.

**Figure 3.**
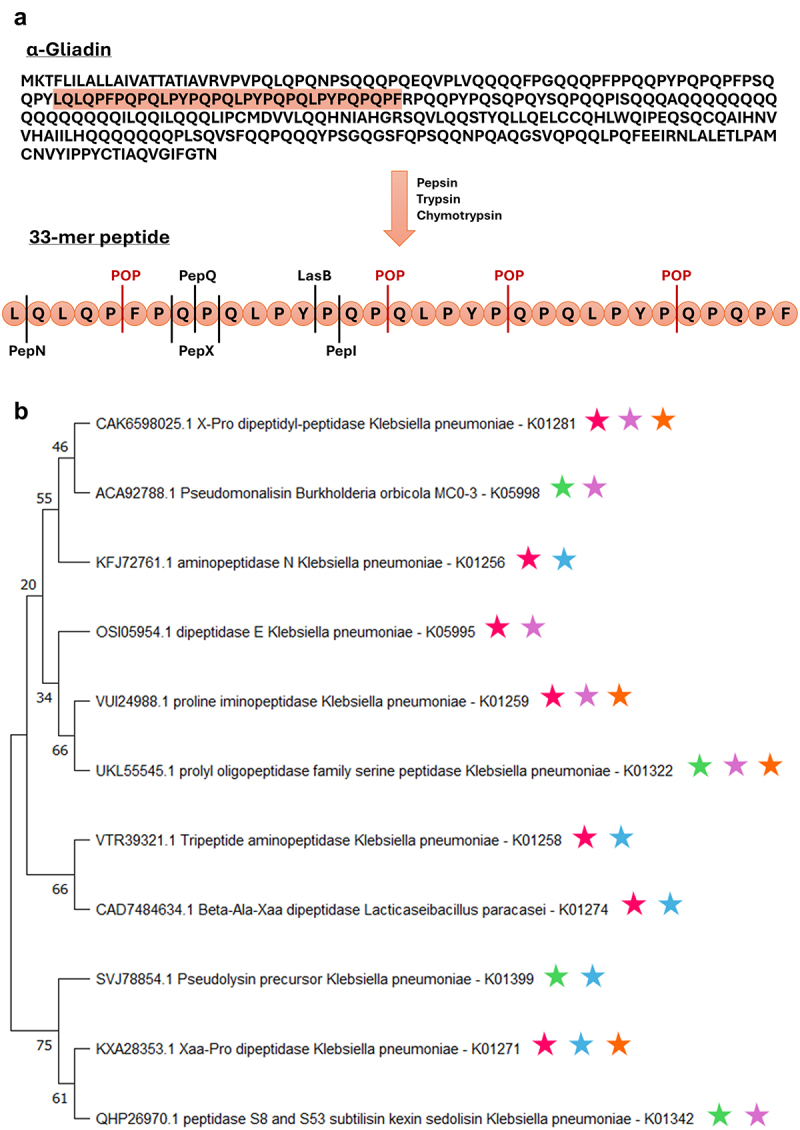
Microbial peptidases capable of digesting gluten peptides. (a) Gluten is a mixture of proteins including α-gliadins which can only be partially digested by human gastric and pancreatic enzymes such as pepsin, trypsin, and chymotrypsin. Large immunogenic peptides, such as the 33-mer peptide, can only be broken down by the concurrent activity of a variety of microbial enzymes. These enzymes can include aminopeptidase (PepN), prolyl oligopeptidase (POP), X-Pro dipeptidyl-peptidase (PepX), Xaa-Pro dipeptidase (PepQ), Pseudolysin (LasB), and proline iminopeptidase (PepI), and some theorized cleavage sites for each peptidase have been indicated. (b) Phylogenetic tree of bacterial derived peptidases, previously reported in the literature to have gluten degrading capacity. Protein sequences were identified from the NCBI protein database, and multiple sequence alignment was performed by ClustalW in MEGA (Version 11.0.13) for the construction of the phylogenetic tree. Exopeptidases are indicated with **red stars**; endopeptidases with **green stars**; serine proteases with **purple stars**; metalloproteases with **blue stars**; and peptidases specific for cleaving proline containing residues are indicated in **orange**.

Human-derived digestive enzymes, including pepsin and trypsin, are not capable of completely breaking down gluten peptides due to the abundance of proline and glutamine.^42,74^ This results in incompletely digested peptides such as the 33-mer immunodominant peptide, which can trigger aberrant immune responses in patients with CeD.^75^ Thus, microbial enzymes capable of cleaving proline bonds are necessary for the digestion of gluten, and their activity can enhance or alleviate the immunogenicity of gluten peptides (Figure 3a).^40,76^

We compared the phylogeny of representative bacterial-derived peptidases reported to have gluten degrading capacity (Figure 3b). Prolyl oligopeptidases (K01322) cleave oligopeptides following proline residues, and their reduction of the immunogenicity of the 33-mer peptide has been previously described.^41^ X-Pro dipeptidyl-peptidases (K01281) release N-terminal dipeptides when the second amino acid is proline and have been shown to work synergistically with prolyl oligopeptidases to reduce the toxicity of the 33-mer peptide.^77^ Alternatively, pseudolysin (K01399) is an elastase that can hydrolyze gluten peptides but has been reported to generate immunogenic peptides that are better able to cross the intestinal epithelial barrier.^76,78^ Aminopeptidase N (K01256) releases N-terminal amino acids including proline; however, the reaction is slower for proline.^77,79^ Xaa-Pro dipeptidase (K01271) breaks apart dipeptides when proline is present at the C-terminus,^77,80^ whilst dipeptidase E (K05995) cleaves N-terminal dipeptides into individual amino acids when aspartate is in the first position.^81^ Finally, proline iminopeptidase (K01259) releases proline from di- and tripeptides where proline is present at the N-terminus.^77^ These peptidases can be expressed by a variety of genera which are capable of surviving on gluten as the sole nitrogen source including *Pseudomonas*, *Lactobacillus*, and *Bifidobacterium*.^25,82^ The combined activity of a variety of microbial peptidases is required for the digestion of gluten, and possible cleavage sites have been shown in Figure 3a.

We observed that serine proteases including dipeptidase E (K05995), proline iminopeptidase (K01259), and prolyl oligopeptidase (K01322) clustered closely, whilst metalloproteases like tripeptide aminopeptidase (K01258) and beta-Ala-Xaa dipeptidase (K01274) tended to cluster together separately (Figure 3B). The phylogenetic tree segregated initially into two distinct nodes, and it is notable that the only peptidase identified as significantly altered in FD from the Shanahan et al., and Shah et al. dataset, Xaa-Pro dipeptidase (K01271), is present on a separate node and thus most distantly related to the significantly altered peptidases in CeD patients. Meanwhile, of the two significantly altered peptidases in NCGS, pseudolysin (K01399) is more closely related to Xaa-Pro dipeptidase (K01271) and subtilisin (K01342), whilst proline iminopeptidase (K01259) is clustered more closely to prolyl oligopeptidase (K01322) and other CeD associated peptidases. Of the included peptidases, four specifically cleave proline-containing residues, including proline iminopeptidase (K01259) altered in NCGS; Xaa-Pro dipeptidase altered in FD (K01271); and X-Pro dipeptidyl-peptidase (K01281) and prolyl oligopeptidase (K01322) altered in CeD. As the proline-rich nature of gluten makes the peptides largely resistant to host derived enzymatic digestion, the changes to these bacterial-derived enzymes are likely important.

## 4. Discussion

This study aimed to collate information on the microbiota of CeD, NCGS, and FD to compare their structure, diversity, and function. Patients with each of these conditions experience meal-related symptoms, especially those triggered by gluten-based foods. As the microbiota can influence immune responses to food, we wished to explore the potential for a microbial signature for GRDs. However, the composition of the microbiota varied significantly between studies and diseases, and this was likely due to the heterogeneity of the sampling sites and sequencing techniques.

The diversity of the microbiota in CeD patients was observably impacted by sampling sites as well as whether patients were adhering to a gluten-free diet. The oral microbiota of CeD patients had a reduced alpha diversity compared to controls, whereas reports on fecal and duodenal alpha diversity were inconsistent. In NCGS patients, however, it was consistently observed that the alpha diversity did not differ from controls and, in FD, changes to alpha diversity were only observed in some studies within the duodenum. Beta diversity in individuals with CeD exhibited a comparable likelihood of mirroring controls as it did of being significantly different from them, across all sampling sites. The beta diversity was not altered in the duodenum of NCGS patients, but findings were inconsistent within stool. In FD, however, an altered beta diversity was described in numerous studies. The effect of disease on measures of microbiota diversity was highly inconsistent across studies, suggesting that overall diversity changes may be less important than specific changes to the microbiota in identifying the disease phenotype.

The microbiota is crucial for appropriate digestion of gluten within the gastrointestinal tract, and numerous species have been identified in the literature that can breakdown gluten. When looking at individual taxa in this systematic review, the majority of studies described significant changes to the abundance of various microbes. However, Nylund et al.,^60^ and Nistal et al.,^58^ did not identify any significant differences in bacterial abundance associated with CeD disease. Nistal et al.,^58^ did however report a non-significant increase in duodenal *Lactobacillus* in CeD which mirrored the findings of Bodkhe et al.,^43^ Furthermore, Tian et al.,^67^ described higher *Lactobacillus* spp. in the saliva of CeD patients, whilst Shi et al.,^66^ reported altered fecal microbiota of CeD. Alternatively, in FD, Cervantes et al.,^44^ saw a reduction in *Lactobacillus* in the gastric microbiota. *Lactobacillus* species have been shown to efficiently degrade gluten in vivo,^25^ and a reduced *Lactobacillus* presence could impair breakdown of gluten. Similarly, the genus *Neisseria* was elevated in the duodenum oral microbiota of patients with CeD^45,46,52,62^ and FD.^64,65,70^ However, in the study by Wauters et al.,^68^ Neisseria was reduced in the duodenum and negatively correlated with symptom severity in FD patients. The growth of *Neisseria*, and particularly the species *Neisseria flavescens*, can be supported by gluten as the main protein source, suggesting that some *Neisseria* species have the capability to breakdown gluten.^25^ Certainly, the research by D’Argenio et al.,^46^ has shown that certain CeD *Neisseria flavescens* strains possess the capacity to induce inflammation and metabolic imbalance in in vitro and ex vivo studies.^83^ Thus, the activity of *Neisseria* in FD warrants further investigation.

The genus *Streptococcus* appears to be largely reduced in the oral and duodenal microbiota of CeD patients.^43,52,57,62^ However, within the stool, Shi et al.,^66^ reported increased *Streptococcus* genus and Francavilla et al.,^49^ found higher *Streptococcus sanguinis* species in CeD patients compared to controls. In FD patients, Shah et al.,^64^ and Shanahan et al.,^65^ described reduced *Streptococcus* in the duodenum. Meanwhile, Fukui et al.,^50^ and Kim et al.,^70^ instead reported that *Streptococcus* was elevated in the mucosa of FD patients. When compared to FD patients, NCGS patients had increased duodenal *Streptococcus* in the study by Shah et al.^64^ *Streptococcus* is a Gram-positive genus of bacteria, most prevalent in the oral cavity, with many species including *Streptococcus sanguinis* having been reported to have glutenase activity.^25,84,85^ A shift in *Streptococcus* localization within the gastrointestinal tract may impact the digestion of gluten, increasing its immunogenicity. *Bifidobacterium* is another commonly altered genus in CeD and FD patients. Specifically, *Bifidobacterium longum* was lower in the stool of CeD patients, as reported by Francavilla et al., and Palmieri et al., but higher in the stomach of FD patients in Igarashi et al. Interestingly, whilst *Bifidobacterium longum* reported gluten-degrading activity,^25^ when used as a probiotic formulation, it can ameliorate inflammation in mouse models of colitis.^86^ Our findings revealed only modest parallels among the gastrointestinal microbiota of FD, NCGS, and CeD, likely due to the considerable diversity in sample sites and sequencing methodologies employed. Nevertheless, evidence of changes to bacterial genera, with known gluten digesting capacity highlighting the likely involvement of the microbiota in the development of GRDs.

16S rRNA microbiota sequencing approaches help reduce some biases inherent in targeted microbiota analysis techniques, such as qPCR and culture-based methods. However, 16S sequencing lacks species-level sensitivity, and biases can be introduced by the choice of primers targeting different gene variable regions. Within this systematic review, at least five distinct variable regions were amplified for sequencing. Another key source of variation in microbiota sequencing is the bioinformatic pipeline and database used to analyze sequences. While various tools and packages are available to aid microbiome analysis, no gold standard method has been established, and techniques like rarefaction may introduce bias.^87^ To mitigate this variability, we accessed raw data to apply a consistent approach to analyzing microbiota sequence data from various sources. However, this was challenging as many articles did not have sequence data deposited in publicly available databases. Public availability of microbiome sequence data has become a standard requirement at publication to ensure transparency and allow researchers to reproduce, validate, and build on findings.^88^ The results of our uniform analysis exemplify the challenges in interpreting microbiome data, as they include numerous results which differ from the published findings. For example, Bodkhe et al., described that ASVs belonging to *Helicobacter* and *Megasphaera* were elevated in CeD compared to controls; however, our analysis of differential abundance did not identify these taxa. Some findings were consistent with published articles, however, including an enrichment of *Actinobacillus* in NCGS patients compared to controls in data from Garcia-Mazcorro et al. Furthermore, we attempted to differentiate disease states based on the microbiota using supervised machine learning with random forest classifiers but found the microbiota to be a poor discriminator of disease state. We were unable to combine datasets for the re-analysis due to differences in sequencing methods and batch effects, and as a result, the small individual sample size of each study likely impacted the ability to differentiate between cases and controls. Despite using consistent methods to analyze microbiota data, we did not identify distinct microbial trends which linked CeD, NCGS, and FD.

Whilst no specific taxa were identified in this study which distinguished GRDs, the role of the microbiota should not be discounted. It is possible that the function of the microbiota is more important than the specific taxa which are present, considering the important role the microbiota plays in digestion of gluten, amongst other foods. Shotgun metagenome sequencing, whilst more expensive than 16S approaches, overcomes many of its caveats and provides a detailed view of microbial functions. Only two of the included articles utilized this methodology; however, we suggest that future studies should look toward metagenomic sequencing as the gold standard. A number of included studies reported on the functional capacity of the microbiota; however, only Bodkhe et al.,^43^ recognized a change in genes related to gluten hydrolysis, with Xaa-pro dipeptidase being reduced in CeD compared to controls. When re-analyzing raw datasets, we looked specifically at the predicted relative abundance of microbial genes related to gluten digestion. It was evident that the genes for numerous peptidases related to the breakdown of gluten were altered in CeD, NCGS, and FD patients when compared to controls. In particular, peptidases with specificity for cleaving proline containing residues were altered in each condition. Gluten is rich in proline and glutamine, making it resistant to host-derived enzymes; thus, we are reliant on bacterial enzymes to cleave proline within dietary gluten.^41,89^ Given that we identified changes to proline hydrolyzing peptidases, it is possible that gluten is differentially digested by the microbiota in these FD, CeD, and NCGS, when compared to controls.

Improper digestion of gluten by gastrointestinal microbes may promote the immunogenicity of gluten and lead to an enhanced host immune response following antigen presentation.^76^ Gluten is only partially digested by gastric and pancreatic enzymes following consumption, and microbial peptidases are largely responsible for breaking down gluten-derived peptides. However, the efficiency of this process and the structure of the resultant peptides is highly variable between different peptidases and microbes, and this can affect the likelihood of these peptides to produce an inappropriate response.^90^ A large variety of T cell epitopes from gluten have been characterized, which can activate CD4+ T cells from CeD patients, and altered peptidase activity can modulate the accessibility of these gluten epitopes to immune cells.^91^ A study by Caminero et al., described that the activity of *Pseudomonas aeruginosa*, isolated from CeD patients, produced gluten peptides which promoted activation of T-cells derived from CeD patients.^76^ This process was mediated by the action of the peptidase, pseudolysin, and importantly, the immunogenicity of the gluten peptides could then be reduced by the enzymatic activity of a *Lactobacillus* species isolated from controls.^76^ This study highlighted the capacity for microbes and their peptidases to determine the reactivity of gluten peptides in the context of GRDs. Immunogenic gluten peptides, like those produced from *Pseudomonas aeruginosa* digestion, can translocate the intestinal epithelial barrier, especially in GRDs where permeability is elevated.^92,93^ In this way, microbially digested gluten can be processed by antigen-presenting cells to initiate innate or adaptive immune responses resulting in symptoms. In CeD, immune activation leads to chronic inflammation and tissue destruction within the small intestine, resulting in intestinal and extra-intestinal symptoms.^9^ The mechanism whereby gluten may be driving responses in NCGS and FD has not been well established by comparison with CeD. However, evidence of elevated duodenal eosinophils in NCGS and FD patients may indicate a subtle allergy-like immune response, which could contribute to subtle inflammation, disrupted epithelium, and symptoms including visceral hypersensitivity.^94^

Given that improper gluten breakdown may amplify immune responses and that this systematic review observed that microbial peptidases involved in gluten digestion are likely altered in CeD, NCGS, and FD, targeting gluten-degrading peptidases presents a potential therapeutic strategy for managing GRDs. Peptidase treatment has been explored in CeD and NCGS, especially as a supportive therapy alongside a gluten-free diet.^42^ However, enzymatic activity can both enhance and reduce the immunogenicity of gluten peptides, posing a significant challenge.^42^ Proline-specific endopeptidase treatment can reduce the amount of immunogenic gliadin peptides translocating through CeD duodenal mucosa in ex vivo experiments.^95^ Furthermore, treatment with a prolyl endopeptidase derived from *Aspergillus niger* significantly enhanced the gastric digestion of gluten in a cohort of healthy participants^96^ and improved symptoms in CeD patients on a long-term gluten-free diet.^97^ A recent review has outlined numerous Phase II clinical trials utilizing a combination of two gluten-degrading peptidases, endoprotease B, and a prolyl endopeptidase derived from *Sphingomonas capsulata*, in the management of CeD.^98^ This formulation has been shown to effectively digest gluten and has been reported to have some efficacy in protecting against small-intestinal mucosal damage induced in CeD patients following consumption of gluten.^99^ In contrast, proline-specific endopeptidase treatment did not improve symptoms in patients with NCGS on a gluten-containing diet.^100^ Additionally, there is very limited evidence for the efficacy of currently available commercial gluten-degrading enzymes.^101^ Despite these challenges, the identification of specific microbial peptidases altered in GRDs from this systematic review provides a new direction for research. Future trials may achieve greater success in symptom reduction and clinical improvement by targeting these specifically altered peptidases. Furthermore, this work highlights the potential to assess the intestinal microbiome of patients with suspected GRDs to determine their gluten digesting capacity, which could be leveraged for diagnosis and to direct treatment. However, additional robust studies detailing the functional capacity of the intestinal microbiome in GRDs are needed to inform this.

Probiotic therapies could also be considered to improve gluten digestion in patients by increasing the abundance of microbes capable of breaking down gluten into peptides unlikely to induce immune activation. There is currently limited evidence for probiotics improving clinical signs of disease in GRDs; however, systematic reviews of CeD and FD literature have described their potential to improve symptoms in patients.^102,103^ Probiotic strains have largely been chosen for proposed anti-inflammatory properties, but interestingly strains from genera including *Lactobacillus* and *Bifidobacterium* with reported gluten degrading capacity have had some of the greatest reported effects in both FD and CeD.^90,103,104^ Probiotic combinations of *Lactobacillus* and *Bifidobacterium* spp. hydrolyzed immunogenic gluten peptides in wheat flour following extended fermentation.^105^ Furthermore, combinations of bacteria from these genera digest gluten peptides in a manner that prevents inflammatory IL-6 production from intestinal epithelial cells in vitro, compared to gluten digested by enzymes mimicking gastric gluten digestion.^106^ This systematic review identifies various genera altered in GRDs, which could be investigated for their potential probiotic effects or selectively outcompeted by more beneficial gluten-degrading probiotics in future studies, as a supportive therapy alongside gluten-free diets. An important question in microbiome research is whether observed microbial changes drive disease pathogenesis or arise as a consequence of the disease itself and its management, particularly the gluten-free diet in GRDs. This systematic review primarily examines baseline microbiomes, prior to any intervention or follow-up, limiting causal inferences. However, in the studies included in our re-analysis, where altered gluten-degrading peptidases were identified, all participants were consuming a standard gluten-containing diet at the time of sampling. This suggests that microbial differences in gluten metabolism are associated with disease rather than dietary status. Future research should explore longitudinal and mechanistic studies to clarify whether microbiome alterations contribute to GRD development or result from disease-related changes.

Whilst a key strength of this study was the re-analysis of published datasets with a new question in mind, we were unable to gain access to the sequence data and associated metadata from all of the identified studies examining the duodenal microbiota by 16S rRNA amplicon sequencing. Thus, a limitation of this re-analysis was that the sample size was too small to draw confident conclusions on how the microbiota compares between conditions or to justify meta-analysis. The inconsistency in diet reporting was a further limitation in our ability to analyze these data. While papers covering CeD and NCGS acknowledged adherence to gluten-free diets, there was limited detail on the composition of the patient diets overall. Similar to FD studies, limited data were presented on participant diet composition. One study did analyze the impact of diet on the duodenal mucosal-associated microbiome obtained via biopsy and reported that the microbiome was not impacted by long-term dietary intake.^65^ However, given the impact of dietary intake on the microbiota,^107^ we propose dietary evaluation as a standard for microbiome studies. Medications can also significantly influence the composition of the microbiome, and the majority of included articles excluded participants who had been taking antibiotics prior to sampling for this reason. Numerous articles also excluded people taking PPIs; however, one study focused on how PPI therapy can impact the microbiome of patients with FD.^68^ They observed that long-term PPI therapy reduced the richness of the microbiome in FD patients and altered specific genera, highlighting the importance of considering medications when analyzing microbiome data. This systematic review underscores the importance of including comprehensive metadata in microbiome studies to allow for appropriate interpretation of results and to enhance future re-analysis of publicly available datasets.

This systematic review has collated the available literature on the gastrointestinal microbiota of patients with CeD, NCGS, and FD to explore the role of microbiota in conditions of gluten hypersensitivity. This study highlighted the heterogeneity of microbiota analysis methods, and the resultant difficulties in interpreting results from different cohorts. Despite methodological, cohort, and sample heterogeneity, specific differences in microbial taxa, including *Lactobacillus*, *Neisseria*, and *Streptococcus*, were identified across CeD, NCGS, and FD. The observed alterations in these genera, known for their gluten-degrading capabilities, point to the microbiota’s likely involvement in the pathogenesis of these conditions. Furthermore, our re-analysis revealed changes in functional genes related to gluten digestion, particularly those involved in proline hydrolysis, suggesting that differential digestion of gluten by the microbiota may enhance the capacity to trigger immune responses or drive symptoms.

## Supplementary Material

Supplemental Material
